# Silk Fibroin‐Based Hydrogels Supplemented with Decellularized Extracellular Matrix and Gelatin Facilitate 3D Bioprinting for Meniscus Tissue Engineering

**DOI:** 10.1002/mabi.202400515

**Published:** 2025-03-06

**Authors:** Jennifer Fritz, Anna‐Christina Moser, Alexander Otahal, Heinz Redl, Andreas H. Teuschl‐Woller, Karl H. Schneider, Stefan Nehrer

**Affiliations:** ^1^ Center for Regenerative Medicine University for Continuing Education Krems Dr.‐Karl‐Dorrek‐Straße 30 Krems 3500 Austria; ^2^ Austrian Cluster for Tissue Regeneration Donaueschingenstrasse 13 Vienna 1200 Austria; ^3^ Department of Orthopaedics and Traumatology Universitätsklinikum Krems Mitterweg 10 Krems 3500 Austria; ^4^ Ludwig Boltzmann Institute for Traumatology The Research Center in Cooperation with AUVA Donaueschingenstrasse 13 Vienna 1200 Austria; ^5^ Department of Life Science Engineering University of Applied Sciences Technikum Wien Höchstädtplatz 6 Vienna 1200 Austria; ^6^ Center for Biomedical Research and Translational Surgery Medical University of Vienna Spitalgasse 23 Vienna 1090 Austria

**Keywords:** 3D bioprinting, meniscus tissue engineering, silk fibroin

## Abstract

The human meniscus transmits high axial loads through the knee joint. This function is compromised upon meniscus injury or treatment by meniscectomy. 3D printing of meniscus implants has emerged as a promising alternative treatment, as it allows for precise mimicry of the meniscus architecture. In this study, silk fibroin (SF) known for its excellent mechanical properties is used to fabricate hydrogels for 3D bioprinting with infrapatellar fat pad‐derived mesenchymal stem cells (IFP‐MSCs). Extracellular matrix (ECM) derived from bovine menisci and gelatin are added to 10% SF to promote cell adhesion and printability. To examine the mutual influence of cells and biomaterial, experiments are conducted with and without IFP‐MSCs. The cells are found to influence crosslinking, β‐sheet formation, and mechanical strength. Variations between printed and casted hydrogels are identified for cell number, metabolic activity, secondary structure, and mechanical strength. Remarkably, the printed hydrogels with IFP‐MSCs exhibited a compressive Young's modulus of 0.16 MPa, which closely resembled that of human osteoarthritic menisci. After initial low viability, IFP‐MSCs in the casted hydrogels are able to proliferate within the biomaterial. The chondrogenic differentiation medium upregulated the expression of chondrogenic markers in the casted hydrogels, indicating promising prospects for future meniscus tissue engineering (TE).

## Graphical abstract

To engineer meniscus tissue, a combination of silk fibroin, decellularized meniscus extracellular matrix, and gelatin is 3D bioprinted with infrapatellar fat pad‐derived mesenchymal stem cells. Cell addition is found to affect the enzymatic crosslinking and the mechanical properties of the hydrogels, whose Young's modulus matches with human meniscus tissue. Cells in casted hydrogels show superior proliferation and chondrogenic differentiation, suggesting potential for meniscus repair and improvement opportunities of this formulation for 3D bioprinting.

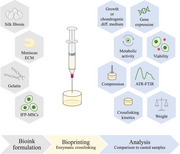

## 1 Introduction

The primary role of the menisci is to transmit load through the knee joint by an increase of the contact area between the femur and tibia. The redistribution of axial compressive load into hoop stresses within the meniscus is caused by its unique combination of material properties, geometry, and ligament attachments.^[^ ^1^ ^]^ These characteristics are achieved by a hydrophilic extracellular matrix (ECM) reinforced with collagen fibers.^[^ ^2^ ^]^ The fibers are particularly responsible for the tensile strength through their specific orientation.^[^ ^3^ ^]^ Upon meniscus injury or treatment by partial or total meniscectomy, the load transmission is compromised.^[^ ^4^ ^]^ Unfortunately, the meniscus integrity loss also contributes to pain, knee instability, and the development of osteoarthritis (OA). Since other conventional treatment approaches for meniscus injuries like repair by suturing and allograft transplants often face limitations, new treatment approaches are highly demanded to effectively preserve the meniscus function.^[^ ^5^ ^]^ 3D printing has emerged as such a promising technology for meniscal TE due to its exceptional precision in controlling scaffold microstructures, critical for imitating the specific collagen architecture of the meniscus.^[^ ^6^ ^]^ Developing a suitable meniscal scaffold necessitates printable materials with mechanical properties that mimic the natural ECM. Additionally, the materials must support cell growth to promote tissue regeneration.

Hydrogels produced from decellularized meniscus ECM were already used as bioink for meniscus TE since they preserve the tissue's complex biochemical composition.^[^ ^7^, ^8^ ^]^ Despite their support of cell growth and differentiation, these scaffolds exhibit poor mechanical properties.^[^ ^7^, ^9^, ^10^, ^11^ ^]^ Therefore, we chose SF extracted from silkworm cocoons as an ideal supplement to increase the mechanical stability. While cell‐laden SF hydrogels have not been used for meniscus 3D bioprinting to our knowledge, the use for cartilage 3D bioprinting was already reported to result in remarkable mechanical properties.^[^ ^12^, ^13^ ^]^ Furthermore, SF hydrogels were already subjected to meniscus 3D printing without cells.^[^ ^14^ ^]^ Among the approaches to crosslink SF, horseradish peroxidase (HRP) catalyzes the formation of di‐tyrosine bonds in combination with free radical formation by hydrogen peroxide (H_2_O_2_).^[^ ^15^ ^]^ Crosslinking SF's tyrosine residues by this mechanism generates a mechanically tunable hydrogel, whose elasticity is dependent on the degree of crosslinking and the environmental conditions.^[^ ^16^, ^17^ ^]^ Moreover, the mild reaction conditions at physiological temperature create optimal conditions for cell encapsulation.^[^ ^16^ ^]^ Mesenchymal stem cells (MSCs) are one of the primary cell types investigated for meniscus TE. One of the sources for MSCs is the infrapatellar fat pad (IFP), which occupies the majority of the anterior knee compartment.^[^ ^18^ ^]^ Arthroscopically harvested IFP‐MSCs were already shown to maintain regenerative potential and viability and have greater proliferation and cartilage differentiation potential than bone marrow MSCs (BMSCs).^[^ ^18^, ^19^ ^]^ In addition, meniscal‐like tissue produced by IFP‐MSCs had higher meniscus gene expression and better mechanical properties than the tissues produced by BMSCs and synovial MSCs.^[^ ^20^ ^]^

It is widely known that biomaterials influence the proliferation and differentiation of included cells.^[^ ^21^, ^22^, ^23^ ^]^ However, less information is reported about the influence of cell addition on biomaterial maturation.^[^ ^24^ ^]^ Usually biomaterial characterization is performed, before cells are added to confirm biocompatibility, although cell embedding affects the hydrogel properties.^[^ ^25^ ^]^ In this study, IFP‐MSCs were included in casted and printed hydrogels with high concentrations of SF and decellularized bovine meniscus ECM to mimic native meniscus tissue. The hydrogels were produced by crosslinking with HRP and H_2_O_2_. To shed more light on the mutual influence of cells and biomaterial, all tests were applied to samples with cells and to cell‐free controls after the initial concentration determination. Particular emphasis was also placed on the differences between printed and casted hydrogels. The overall aim of this work was to develop a SF‐based hydrogel supplemented with ECM, which effectively integrates cells and enables 3D bioprinting for meniscus TE. Furthermore, we anticipated that the bioprinted hydrogels incorporating the novel combination of SF and ECM would promote cell survival and proliferation and provide necessary mechanical properties to mimic meniscus tissue.

## 2 Results

### 2.1 Characterization of Biomaterials

**Figure 1:**
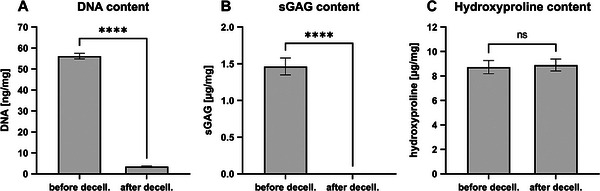
Characterization of bovine meniscus and ECM powder by biochemical assays. A) DNA content reduction by decellularization (*n* = 3). B) Loss of sGAGs (*n* = 3). C) Preservation of collagen represented by hydroxyproline content (*n* = 3).

Bovine meniscus‐derived ECM was used in the bioink to provide collagen for the meniscal construct and to facilitate cell attachment. DNA quantification of the bovine menisci before and after decellularization confirmed the significant reduction of DNA by 94% (**Figure** **1**A). While the sulfated glycosaminoglycans (sGAGs) were lost during decellularization (Figure 1B), the collagen content was preserved with a final hydroxyproline concentration of 8.9 µg mg^−1^ in the ECM (Figure 1C). Although ECM with a high collagen content should facilitate gelation at 37 °C, the storage of a 5% (w/v) ECM solution in 1X PBS at 37 °C at a pH of 7.4 did not lead to gelation. However, it was noticed that the storage at 4 °C induced gelation.

#### 2.1.1 Crosslinking Kinetics of Biomaterials

**Figure 2:**
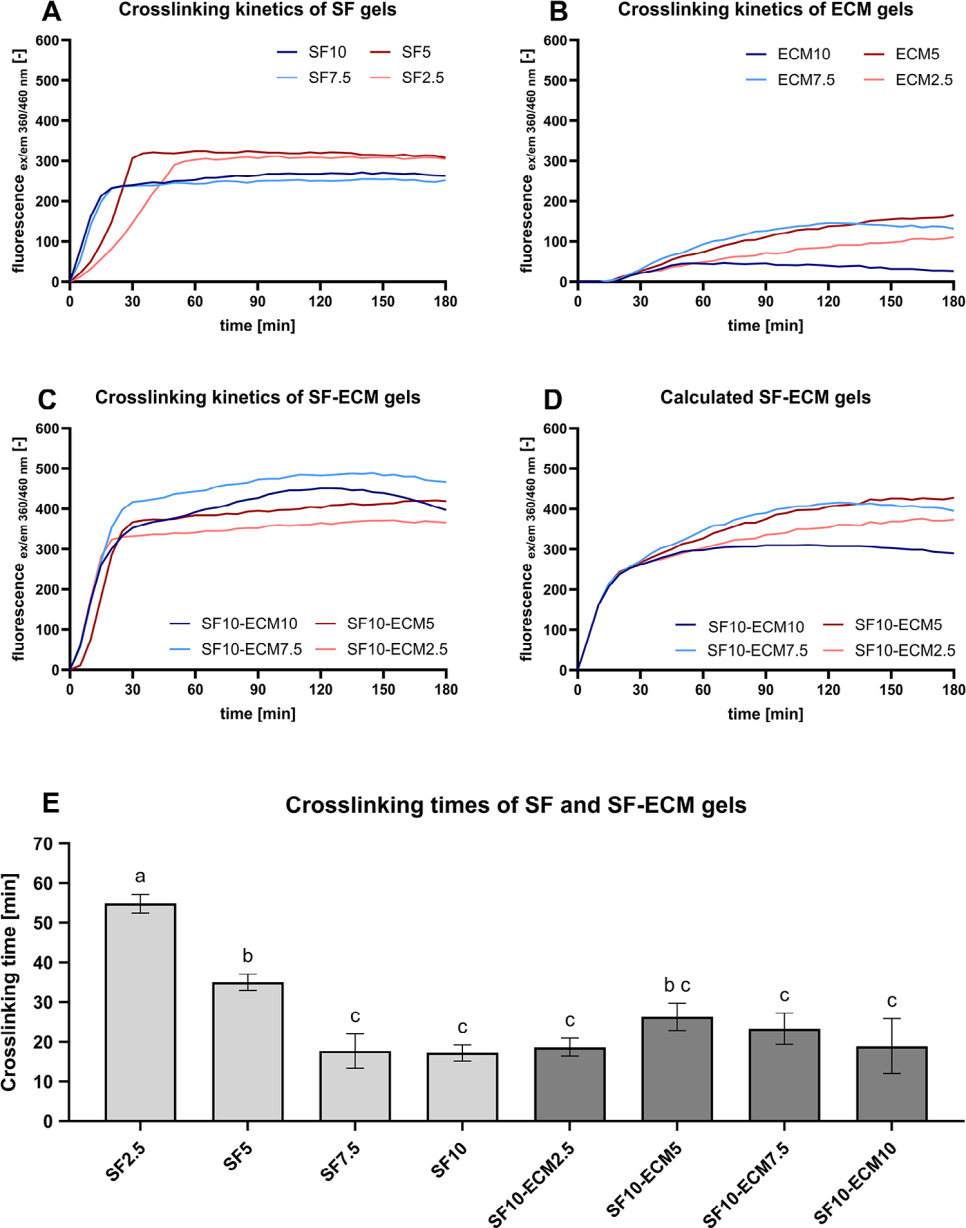
Crosslinking kinetics of SF‐based or ECM‐based hydrogels with 10 u mL^−1^ HRP and 0.01% H_2_O_2_ at 37 °C. The means of three technical replicates are illustrated. A–C) Crosslinking kinetics of hydrogels with varying SF and ECM concentrations (*n* = 1). D) Combined fluorescence by addition of the individual fluorescence values of SF10 and ECM2.5‐ECM10 for comparison with measured combinations (*n* = 1). E) Crosslinking times of SF and SF‐ECM gels. Data were compared using one‐way ANOVA with Tukey post‐hoc test. Different letters above the bars (a–j) indicate statistically significant differences between the groups (*p* < 0.05). Groups sharing a letter are not significantly different (*p* ≥ 0.05) (*n* = 1).

To determine the ideal SF and ECM concentration for fast crosslinking, the crosslinking kinetics of single and combined components were monitored via fluorescence analysis of di‐tyrosine crosslinks formed by HRP and H_2_O_2_. To eliminate the effect of the initial fluorescence of amino acids, background subtraction was applied with the first fluorescence value. The higher SF concentrations of 10% and 7.5% with 13‐ and 15‐min crosslinking time reached the fluorescence plateau significantly earlier than the lower concentrations of 5% and 2.5% (**Figure** **2**E). Although higher SF concentrations led to faster crosslinking, the end fluorescence value of the lower concentrations was higher, which indicates that more crosslinks were formed despite the lower availability of tyrosine (Figure 2A). An initial drop of fluorescence in the first 10 min was observed for the samples only containing ECM (Figure 2B). Therefore, the third value was used for background subtraction in this case. In general, the application of HRP and H_2_O_2_ for crosslinking of 2.5–10% ECM failed to induce enough crosslinking for stable gel formation. As presented in Figure 2E, the combination of 10% SF with 2.5–10% ECM led to crosslinking times of 18–25 min. The curves of 10% SF were added to the ones of 2.5–10% ECM and compared to the measured combination to highlight potential interactions between SF and ECM. The calculated combined fluorescence resulted in lower fluorescence values for 10% and 7.5% ECM than actually measured (Figure 2D). The fluorescence drop of the ECM samples was further investigated in the supplements (Figures S2 and S3, Supporting Information).

### 2.2 Crosslinking Kinetics with IFP‐MSCs

**Figure 3:**
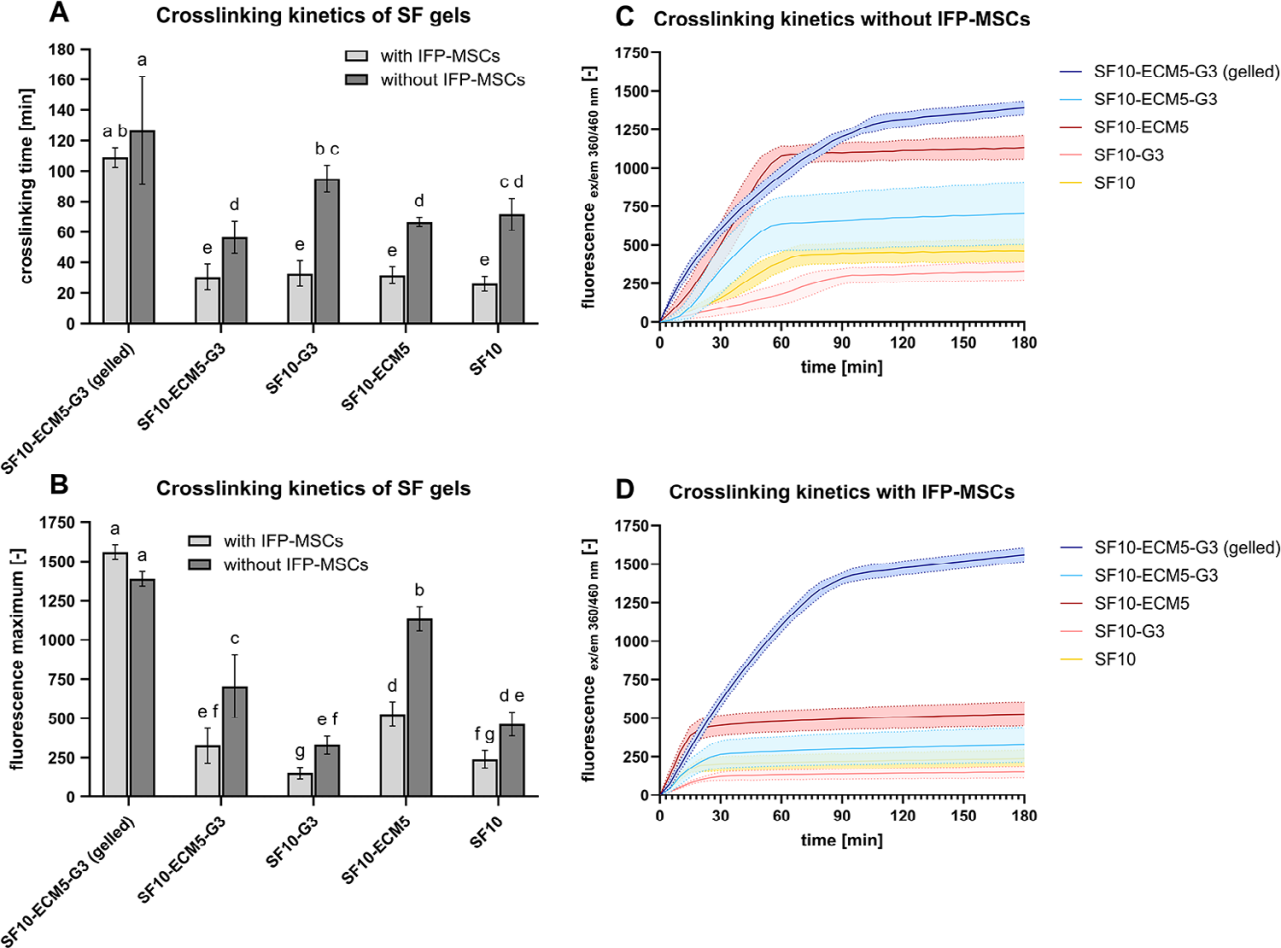
The influence of IFP‐MSCs on SF‐based hydrogel crosslinking using 10 u mL^−1^ HRP and 0.01% H_2_O_2_ at 20 °C. A) Crosslinking times of SF‐based hydrogels with and without IFP‐MSCs. Data were compared using two‐way ANOVA with Tukey post‐hoc test. Different letters above the bars (a–g) indicate statistically significant differences between the groups (*p* < 0.05). Groups sharing a letter are not significantly different (*p* ≥ 0.05) (*n* = 3). B) Fluorescence maxima of SF‐based hydrogels (*n* = 3). C) Crosslinking kinetics of SF‐based hydrogels without cells (*n* = 3). D) Crosslinking kinetics of SF‐based hydrogels with 10^6^ IFP‐MSCs/mL (*n* = 3).

To determine whether the presence of cells altered the crosslinking process, fluorescence analysis was performed with IFP‐MSCs. The formation of di‐tyrosine crosslinks was represented by the fluorescence increase until a plateau. Porcine skin gelatin type A (G) was added as an alternative gelling agent to ECM. The approach of 3D bioprinting with subsequent crosslinking initiation by H_2_O_2_ addition was modeled by adding H_2_O_2_ after gelation for the condition SF10‐ECM5‐G3 (gelled). All other conditions were crosslinked immediately after material mixture to represent the casted hydrogels of the study. Comparing the graphs without IFP‐MSCs (**Figure** **3**C) and with IFP‐MSCs (Figure 3D), the curves of the corresponding conditions almost overlapped in the beginning. However, after the initial steep increase the fluorescence of the samples with IFP‐MSCs reached a plateau earlier than without IFP‐MSCs. This indicates that less crosslinks were formed with IFP‐MSCs.

The differences were further investigated by calculating the mean crosslinking times and the fluorescence maxima (Figure 3A,B). Cell addition significantly reduced the crosslinking times and the fluorescence maxima for all conditions except for SF10‐ECM5‐G3 (gelled). The conditions without previous gelation were all crosslinked within 45 min, when IFP‐MSCs were added. In contrast, crosslinking required up to 100 min, when no IFP‐MSCs were added. The previous gelation increased the crosslinking time up to 165 min, with the means being 127 min without cells and 109 min with cells. Interestingly, ECM addition to cell‐free samples significantly increased the fluorescence maxima, while G addition significantly decreased it in comparison to SF10 (Figure 3B).

### 2.3 3D‐Printing of Hydrogel Formulations

**Figure 4:**
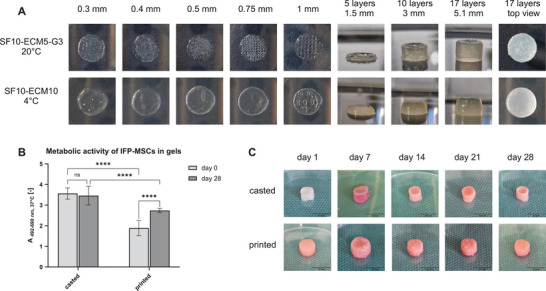
3D (bio)printing SF10‐ECM5‐G3 in comparison to casted gels. A) Printability test of SF10‐ECM5‐G3 at 20 °C and SF10‐ECM10 at 4 °C without crosslinking. B) Metabolic activity of IFP‐MSCs in SF10‐ECM5‐G3 gels (*n* = 1, with three technical replicates). C) Casted and printed cell‐free controls after crosslinking with HRP and H_2_O_2_.

**Figure 5:**
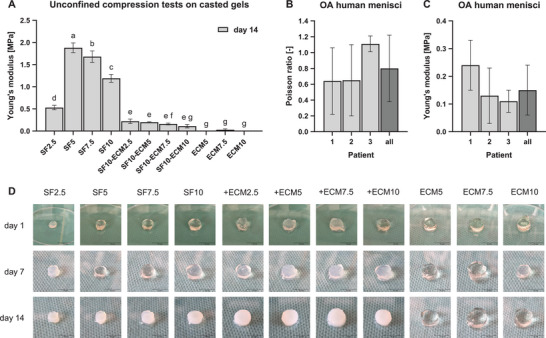
Mechanical properties of hydrogels and OA human menisci. The means of three technical replicates are illustrated. A) Compressive Young's modulus of hydrogels on day 14 was calculated from unconfined compression tests at 10% strain. Data were compared using two‐way ANOVA with Tukey post‐hoc test. Different letters above the bars (a‐g) indicate statistically significant differences between the groups (*p* < 0.05). Groups sharing a letter are not significantly different (*p* ≥ 0.05) (*n* = 1). B) Poisson ratio of OA human menisci (*n* = 3). C) Young's modulus of OA human menisci at 10% strain (*n* = 3). D) Transparency change caused by β‐sheet formation. (scale bar = 10 mm).

As described in Figure S5 (Supporting Information), the dimension measurements showed that SF10 gels kept their shape and weight better than gels with lower SF concentrations. Additionally, increasing SF concentrations decreased the Young's modulus (Figure 5A). However, only the addition of ECM required for cell instruction decreased Young's modulus to values obtained in the testing of human menisci. Since lower ECM concentrations in combination with 10% SF led to more stable gels in regard of height, diameter, and weight (Figure S5, Supporting Information), 5% ECM was chosen as the best concentration. However, in the printing trials 3% G had to be added to achieve printability at 20 °C. Nevertheless, printing without G was tested by increasing the ECM concentration to 10% for sufficient gelation at 4 °C. SF10‐ECM5‐G3 and SF10‐ECM10 were extruded in the form of grids with a diameter of 8 mm and spacings between 0.3 to 1 mm (**Figure** **4**A). With SF10‐ECM5‐G3, the individual strands were already clearly separated with a grid spacing of 0.5 mm, while 1 mm spacing for SF10‐ECM10 still resulted in partly merged strands. When several layers with a grid spacing of 0.5 mm were printed on each other, SF10‐ECM5‐G3 led to stable cylinders with a straight shell, where the individual layers were still recognizable. With SF10‐ECM10, the cylinder shell was curved, and all layers were merged. Considering the smaller possible grid spacing and the more stable cylinders, the printability of SF10‐ECM5‐G3 exceeded the one of SF10‐ECM10. Therefore, SF10‐ECM5‐G3 was used for the following studies.

### 2.4 3D‐Bioprinting and Casting with IFP‐MSCs

3D‐(bio)printed samples with and without IFP‐MSCs were compared to casted ones to evaluate effects of extrusion with a 3D‐printer. While the shape of the casted and printed cell‐free controls was visually similar over time, the printed gels were already opaque 1 day after printing with consequent crosslinking and the casted gels kept their transparency until day 7 (Figure 4C). On day 7, the top of the casted gels also started to turn opaque and the whole gels were opaque on day 14.

#### 2.4.1 Mechanical Properties of Gelled Biomaterials

The compressive Young's modulus of the hydrogels was calculated to identify the concentrations that approximate the mechanical properties of the natural meniscus as closely as possible. The Young's modulus of all pure SF samples and SF10‐ECM samples (except for SF10‐ECM10) was significantly higher on day 14 compared to day 1. As no samples had Young's modulus higher than 0.04 MPa on day 1 and day 7, the focus was laid on day 14. Within the pure SF samples, SF5 had the highest Young's modulus with 1.88 MPa. Adding more SF to the hydrogel (SF7.5 and SF10) significantly decreased the Young's. Adding any ECM concentration to 10% SF significantly reduced Young's modulus to below 0.23 MPa (**Figure** **5**A). The Young's modulus increase over time also coincided with a transparency change caused by β‐sheet formation.^[^ ^26^ ^]^ While all hydrogels were transparent on day 1, all SF‐containing hydrogels became turbid until day 14 (Figure 5D).

The mechanical properties of OA menisci were determined to compare them to the mechanical properties of the tested hydrogels. The compression tests resulted in a mean Young's modulus of 0.15 MPa ± 0.09 (SD) and a mean Poisson ratio of 0.8 ± 0.42 (SD). No significant differences were observed between the samples of three patients. However, not only the variability between the different patient samples but also within the samples of each patient was very high (Figure 5B,C).

#### 2.4.2 Metabolic Activity of IFP‐MSCs

Since the biocompatibility of the materials was only analyzed for OA chondrocytes in casted gels so far (Figure S4, Supporting Information), the metabolic activity of IFP‐MSCs of one donor was evaluated in casted and printed gels on day 0 and 28 to corroborate the biocompatibility for future applications. In the casted gels, the metabolic activity was maintained from day 0 to day 28 (Figure 4B). In the printed gels, the metabolic activity significantly increased from day 0 to day 28. However, it was significantly lower than in the casted gels.

#### 2.4.3 Secondary Protein Structures

**Figure 6:**
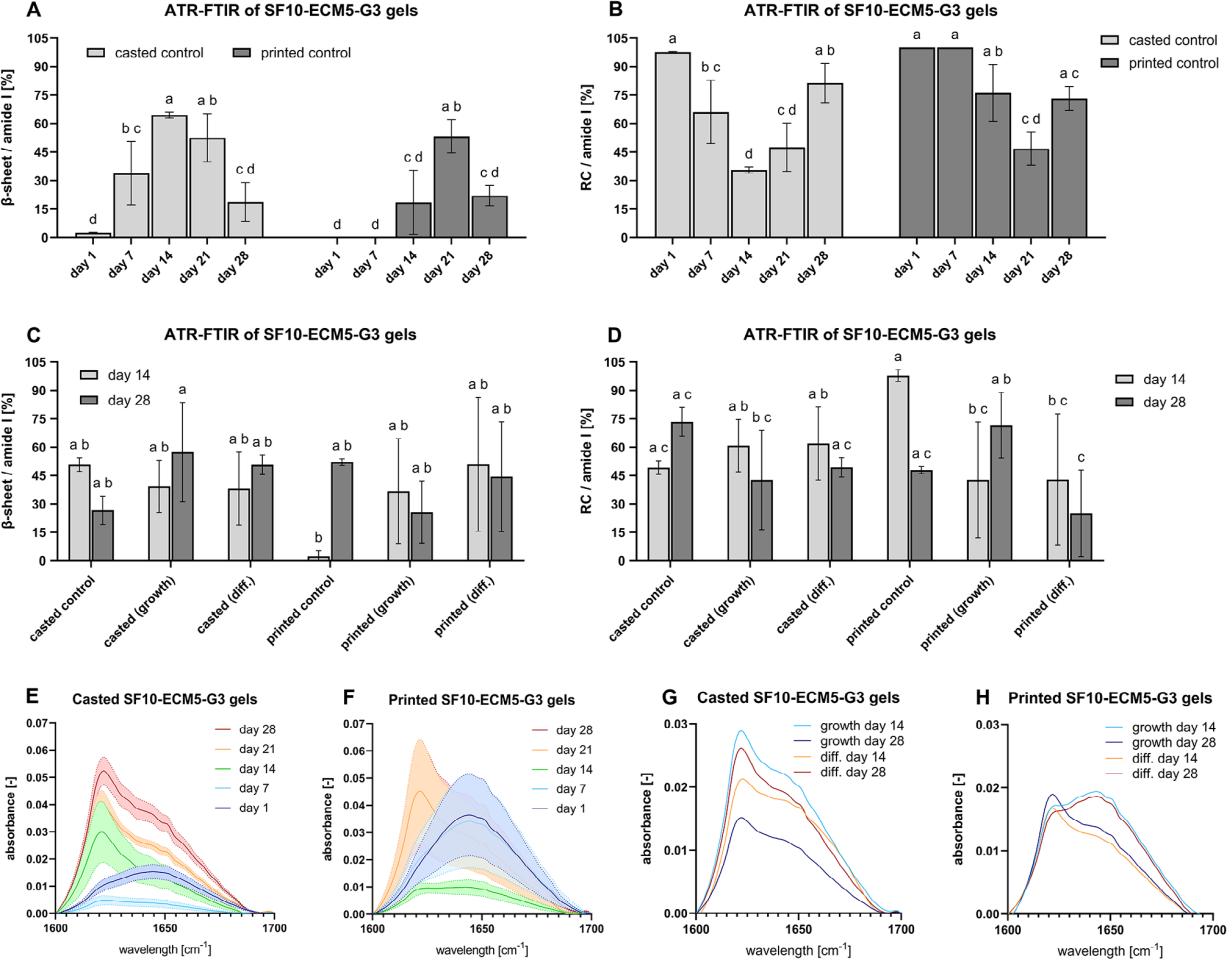
Secondary structure changes caused by 3D (bio)printing. The means of three gels as technical replicates were illustrated for the cell‐free controls. A) β‐sheet content of casted and printed cell‐free controls in 1X PBS. Data were compared using two‐way ANOVA with Tukey post‐hoc test. Different letters above the bars (a–d) indicate statistically significant differences between the groups (*p* < 0.05). Groups sharing a letter are not significantly different (*p* ≥ 0.05) (*n* = 1). B) RC content of casted and printed cell‐free controls in 1X PBS (*n* = 1). C) β‐sheet content of casted and printed gels with IFP‐MSCs in comparison to cell‐free controls (*n* = 3). D) RC content of casted and printed gels with IFP‐MSCs in comparison to cell‐free controls (*n* = 3). E) Amide I region in casted cell‐free controls in 1X PBS (*n* = 1). F) Amide I region in printed cell‐free controls in 1X PBS (*n* = 1). G) Amide I region in casted gels with IFP‐MSCs (*n* = 3). H) Amide I region in printed gels with IFP‐MSCs (*n* = 3).

To observe biomaterial maturation over time, relative proportions of β‐sheets and RCs in casted and printed SF10‐ECM5‐G3 gels incubated in 1X PBS were analyzed by attenuated total reflection‐ Fourier transform infrared (ATR‐FTIR) spectroscopy. The casted cell‐free controls showed a low mean fraction of β‐sheets in **Figure** **6**A on day 1 and 7, while the β‐sheet peaks were clearly distinguishable on day 14, 21, and 28 (Figure 6E). However, not only the β‐sheet peak gained in intensity between day 14 and 28 but also the RC peak. Therefore, the β‐sheet fraction significantly decreased from its maximum on day 14 to day 28. The printed cell‐free controls contained no β‐sheets on day 0 and 7 (Figure 6A). The maximum β‐sheet fraction was only achieved on day 21 and significantly decreased to day 28. The β‐sheet fraction of the casted gels was significantly larger on day 7 and 14 compared to the printed gels, whereas the fractions converged again on day 21 and 28 (Figure 6A). In conclusion, β‐sheet formation, which is considered to contribute to brittleness of SF‐based hydrogels, was delayed by 3D printing.^[^ ^27^ ^]^

Assessing the contribution of different media to cell‐free biomaterial maturation, β‐sheet formation was similar in casted hydrogels incubated in growth medium as well as in 1X PBS (Figure 6A,C). The observation that the printed cell‐free controls maintained a majority of RCs until day 14 in 1X PBS was even intensified in growth medium, since the RC fraction still made up 97.79% on day 14 and 47.91% on day 28 (Figure 6D), which was comparable to 46.74% on day 21 in 1X PBS (Figure 6B).

When the IFP‐MSCs of three donors were included into the casted and printed gels, high standard deviations were obtained for the ATR‐FTIR spectra. Therefore, only the means were illustrated in the spectral diagrams (Figure 6G,H). Nevertheless, β‐sheet peaks could be clearly identified in all casted conditions (Figure 6G). In contrast, not all conditions for the printed gels induced clear β‐sheet peaks (Figure 6H). Regardless, the calculated mean β‐sheet fractions in Figure 6C were similar between all conditions containing cells (25.58–50.92%). IFP‐MSCs in the printed samples induced faster β‐sheet formation in comparison to the cell‐free controls (Figure 6C). This behavior could not be observed for the casted samples. In general, IFP‐MSC inclusion eliminated the significant difference found between casted and printed cell‐free controls.

#### 2.4.4 Weight Retention

**Figure 7:**
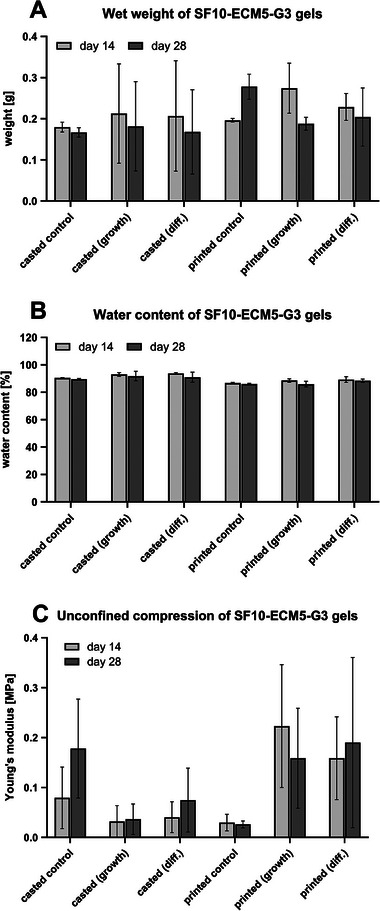
The effect of 3D bioprinting on weight and stability. The IFP‐MSCs were cultivated in casted or printed hydrogels exposed to growth or chondrogenic differentiation medium, while the cell‐free controls were cultivated in growth medium. A) Hydrogel wet weight (*n* = 3). B) Water content calculated with wet and dry weight (*n* = 3). C) Compressive Young's modulus at 10% strain tested by unconfined compression. The only significant difference was found between casted (growth) day 14 and printed (growth) day 14 (*n* = 3).

The hydrogels were weighed on day 14 and 28 to evaluate their stability over time and were weighed again after freeze‐drying to calculate the water content. The casted hydrogels exhibited a higher variability of their wet weight than the printed ones, as the casted samples with IFP‐MSCs from one donor dissolved partly. Although the mean wet weight was always slightly lower on day 28 than on day 14 except for the printed cell‐free controls, no significant difference was observed (**Figure** **7**A). The subsequently calculated water content ranged from to 84.0% to 95.8%, where it was always slightly lower on day 28 than on day 14 (Figure 7B).

#### 2.4.5 Mechanical Properties

The compressive Young's modulus was determined by unconfined compression tests as a measure of hydrogel stiffness. The mean Young's modulus of the casted samples increased over time (Figure 7C). The casted cell‐free controls had a Young's modulus between 0.03 MPa on day 14 and 0.254 MPa on day 28. The addition of IFP‐MSCs to the casted samples reduced the Young's modulus to values between 0.00 MPa on day 14 to 0.18 MPa on day 28. In contrast, the addition of IFP‐MSCs to the printed cell‐free controls increased the Young's modulus from values between 0.01 and 0.05 MPa to values between 0.01 and 0.53 MPa. However, only the highest mean of 0.22 MPa with IFP‐MSCs in growth medium on day 14 was significantly higher than for the casted samples.

#### 2.4.6 Cell Viability

**Figure 8:**
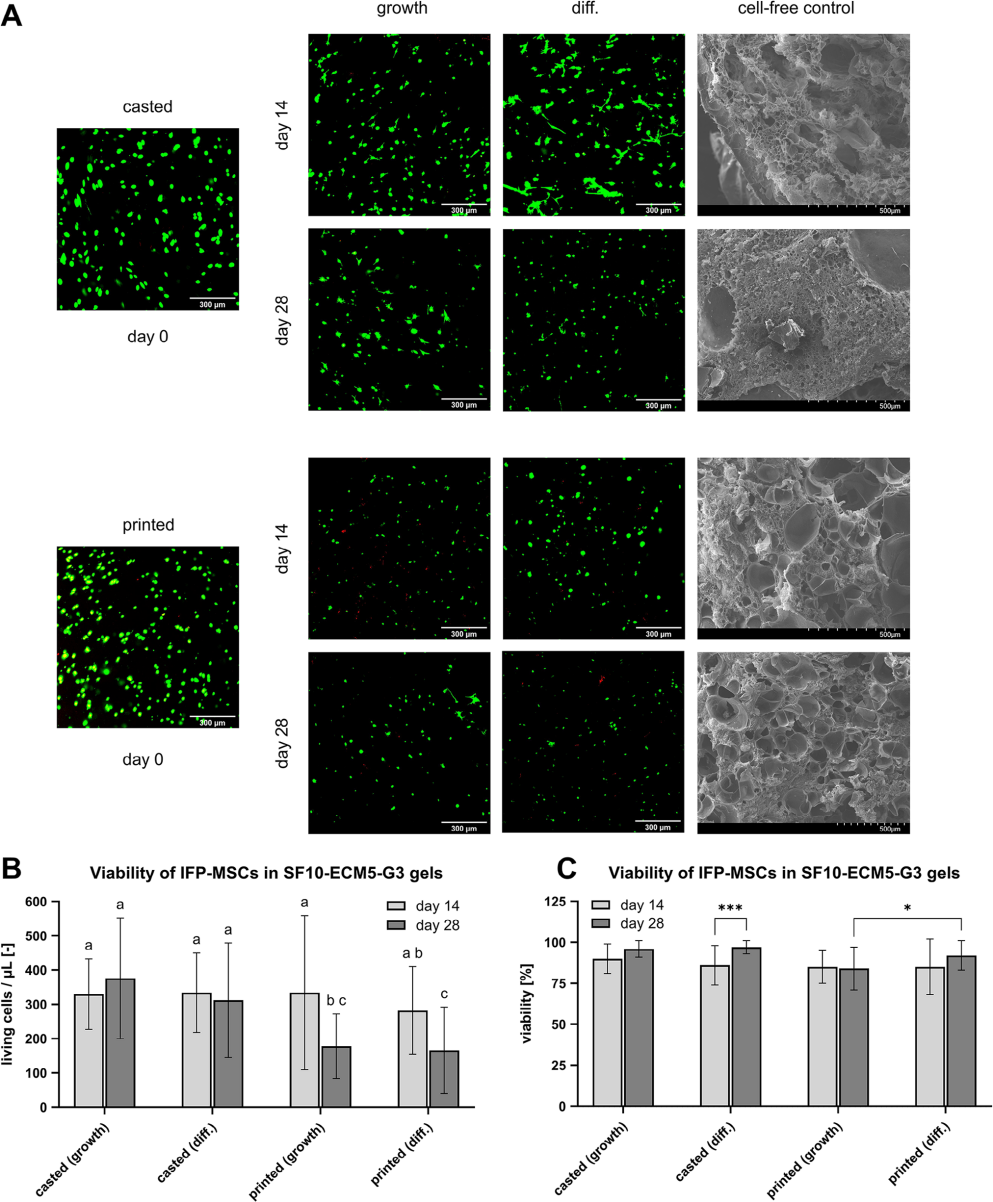
The effect of 3D bioprinting on cell viability. The IFP‐MSCs were cultivated in casted or printed hydrogels exposed to growth or chondrogenic differentiation medium. A) Confocal microscopy images after live and dead staining of hydrogel cross sections (central area) and SEM images of cell‐free controls. The scale indicates 300 µm in the confocal microscopy images and 500 µm in the SEM images (*n* = 1). B) Living cells counted in ImageJ. Data were compared using two‐way ANOVA with Tukey post‐hoc test. Different letters above the bars (a–c) indicate statistically significant differences between the groups (*p* < 0.05). Groups sharing a letter are not significantly different (*p* ≥ 0.05) (*n* = 3). C) Calculated viability (*n* = 3).

Cross sections (Figure S1, Supporting Information) of the casted and printed hydrogels with IFP‐MSCs were examined by confocal microscopy after live and dead staining to evaluate cell viability. The images of the middle of the cross sections were presented in **Figure** **8**A. In addition, the hydrogel structure of the cell‐free controls was visualized by scanning electron microscopy (SEM) to identify pores for cell growth and proliferation. The casted samples with IFP‐MSCs of the shown triplicate on day 0 contained 122 ± 40 (SD) living cells per µL with a low viability of 59% ± 13% (SD). When the samples were incubated in growth medium, the living cell number per µL increased to 271 ± 94 (SD) on day 14 and 238 ± 100 (SD) on day 28 with high viabilities. In differentiation medium, similar cell numbers and viabilities could be observed. Despite the larger pores in the printed cell‐free constructs, the printed samples did not induce more cell proliferation than the casted ones. The printed samples, where 214 ± 69 (SD) living cells per µL were detected on day 0, had a viability of 56% ± 16% (SD). The incubation in growth medium until day 14 led to 383 ± 135 (SD) living cells per µL. Afterward, the living cell number per µL dropped to 254 ± 72 (SD) on day 28. Similar results were obtained for the printed samples in the differentiation medium (Figure 8A).

The living cell numbers and viabilities of the IFP‐MSCs from all three donors and individual triplicates were summarized for days 14 and 28 (Figure 8B). The mean values of living cells per µL in casted samples ranged from 312 to 376. Printing the IFP‐MSCs led to similar living cell numbers per µL of 334 ± 225 (SD) in growth medium and 282 ± 128 (SD) in differentiation medium on day 14. On day 28, significantly lower living cell numbers per µL were obtained for the printed samples in comparison to the casted ones. The mean living cell numbers per µL in the printed samples were also significantly lower on day 28 than on day 14 (Figure 8B).

Although casting the samples partly resulted in significantly higher living cell numbers than printing them, the cell viability was not as strongly affected as the cell number. The mean viabilities for the casted samples ranged from 86% to 97%, while the viabilities for the printed samples ranged from 84% to 92% (Figure 8C). Nevertheless, the casted samples in growth and differentiation medium led to significantly higher cell viabilities than the printed samples in growth medium on day 28. The mean viability was higher on day 28 than on day 14 for all conditions except for the printed samples in growth medium, despite the casted samples in differentiation medium on day 14 and 28 having the only significant difference (Figure 8C).

#### 2.4.7 Gene Expression

**Figure 9:**
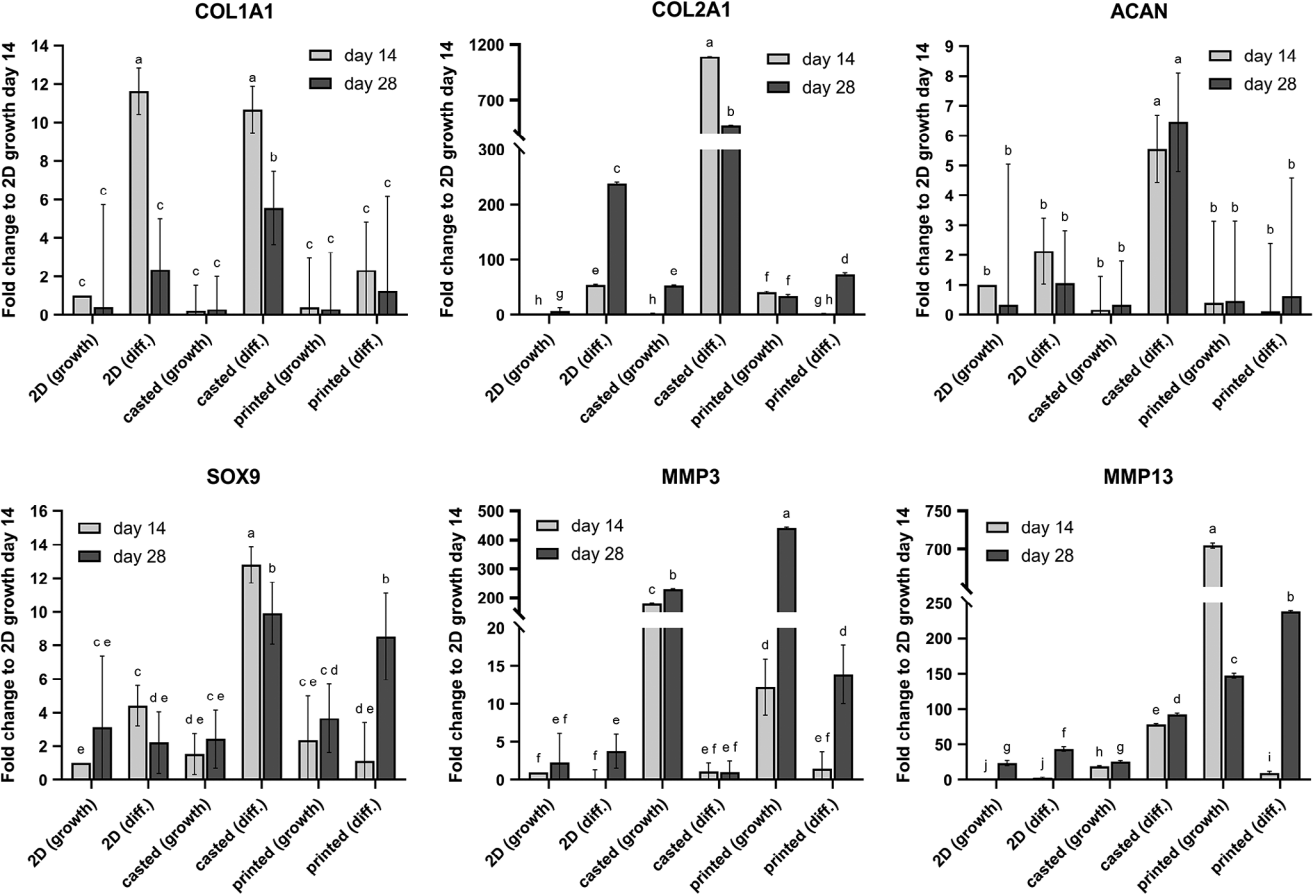
Gene expression of IFP‐MSCs. The IFP‐MSCs were cultivated in 2D, casted, or printed hydrogels exposed to growth or chondrogenic differentiation medium. The Ct values were normalized to GAPDH and to the 2D control in growth medium of day 14 by the 2^−ΔΔCt^ method. Data were compared using two‐way ANOVA with Tukey post‐hoc test. Different letters above the bars (a–j) indicate statistically significant differences between the groups (*p* < 0.05). Groups sharing a letter are not significantly different (*p* ≥ 0.05) (*n* = 3).

The differentiation potential of IFP‐MSCs in casted and printed hydrogels was evaluated by gene expression analysis (**Figure** **9**). All Ct (cycle threshold) values were normalized to GAPDH and to the 2D control in growth medium of day 14 by the 2^−ΔΔCt^ method. In general, application of chondrogenic differentiation medium significantly increased COL1A1, COL2A1, ACAN, SOX9, and MMP13 expression in the casted samples in comparison to growth medium, while MMP3 was significantly downregulated. Especially COL2A1 was remarkably overexpressed, reaching ≈1100‐fold higher levels than the 2D control in growth medium on day 14. Within the 2D controls, COL2A1 was significantly upregulated on both days, while the expression of the other genes was either upregulated or not significantly affected. Within the printed samples, COL1A1 and ACAN expression were not altered by differentiation medium, whereas MMP3 expression was significantly downregulated. SOX9 and COL2A1 expression in the printed samples was only significantly upregulated on day 28. Casted samples cultured in differentiation medium exhibited superior performance, demonstrating significantly higher COL1A1, COL2A1, ACAN, and SOX9 expression, when compared to the printed samples in differentiation medium, the only exception being SOX9 on day 28. The data indicate that casted hydrogels were the most effective in promoting chondrogenic differentiation, thereby approximating the native meniscus.

To assess the intrinsic potential of the hydrogel to upregulate chondrogenic markers independently of exogenous differentiation signals, samples cultured in growth medium were compared. Casting the samples only significantly upregulated COL2A1 on day 28, while the other chondrogenic markers were unaffected in comparison to the 2D controls. However, MMP3 was significantly downregulated on day 14 and day 28, whereas MMP13 was only significantly downregulated on day 14. Comparing the casted samples to the printed ones, COL2A1 expression was only significantly upregulated on day 28. Gene expression of COL1A1, ACAN and SOX9 was unaffected. Downregulation of MMP13 on both days and of MMP3 on day 28 could indicate, that casting contributed less to remodeling than 3D bioprinting.

## 3 Discussion

In this study, a 3D bioprintable SF‐ECM‐G hydrogel was developed for use in meniscus TE. The development uncovered relationships between the modification of HRP/H_2_O_2_ crosslinking by the presence of IFP‐MSCs and the resulting alterations in secondary structure, mechanical properties, cell viability, and differentiation capacity. The alterations, particularly those observed between casted and printed gels, will be discussed in more detail below.

To enable printability, G was added as an alternative gelation agent, since ECM failed to induce gelation at RT or above. Although pure digested collagen was reported to gel by fibril formation dependent on ionic strength, pH, and temperature, the interplay of all components was found to be important in the gelation kinetics of ECM.^[^ ^9^, ^28^ ^]^ For example, the presence of GAGs influences the gelation by electrostatic interaction.^[^ ^9^ ^]^ Therefore, the absence of GAGs shown in Figure 1 may have influenced gelling ability. Since the collagen subunits from pepsin digestion have a melting point of 32 °C, subunits also may have been prevented from self‐assembly at lower temperatures.^[^ ^29^ ^]^ One observation in favor of this hypothesis is that ECM‐induced gelation at 4 °C. Denatured collagen and G are able to undergo a transition from RC to triple helix during cooling facilitating the formation of ordered gel networks.^[^ ^29^, ^30^ ^]^ Although the SF‐ECM gels proved to be inferior to SF‐ECM‐G gels in the printability tests, optimization of the processing temperatures could improve their characteristics in further studies.

The missing ECM gelation was also evident in the crosslinking kinetics measurement in Figure 2. Pure ECM samples led to a small increase in fluorescence after an initial fluorescence drop. The drop could be the result of induced oxidative cleavage upon contact with H_2_O_2_ and HRP and consequent polymer degradation. Indeed, increasing H_2_O_2_ concentrations resulted in increasing fluorescence drops (Figure S2). Nevertheless, ECM fluorescence also decreased slightly without the application of HRP independent of the H_2_O_2_ concentration. This suggests that the experimental conditions further contributed to degradation of collagen, which has a fluorescence emission peak at 440 nm, by temperature or pH reduction by H_2_O_2_.^[^ ^31^ ^]^ While crosslinking ECM, in which collagen type I contains 0.89% tyrosine residues according to literature, proved to be limited, crosslinking SF solutions with ≈5% tyrosine was successful.^[^ ^15^, ^32^ ^]^ Lower SF concentrations (2.5% and 5% SF) led to slower crosslinking and to higher end fluorescence values. Although higher SF concentrations should provide more crosslinking sites, the higher viscosity of the solutions and the faster crosslinking might have inhibited the formation of more crosslinks. When 10% SF was supplemented with 2.5–10% ECM, the crosslinking kinetics were dominated by SF. The observation of the combination led to slightly higher fluorescence end values than the theoretical sum of the individual fluorescence curves. Based on these findings, it is plausible that the SF crosslinking reaction either partially attenuated the initial ECM fluorescence reduction or facilitated supplementary crosslinking between SF and ECM.

When IFP‐MSCs were included, the crosslinking time and crosslinking intensity reported as fluorescence maxima significantly decreased for all conditions in Figure 3 except for the previously gelled SF10‐ECM5‐G3. This finding suggests that IFP‐MSCs interfered with the crosslinking mechanism, when they were mixed with the crosslinking agents. Cells were previously shown to reduce crosslinking by the capture of free radicals and the inactivation of H_2_O_2_.^[^ ^24^ ^]^ Interestingly, the crosslinking kinetics were not significantly altered, when IFP‐MSCs and HRP were gelled before H_2_O_2_ addition, which suggests that H_2_O_2_ would not be inactivated by the cells for the 3D bioprinting approach. The lack of H_2_O_2_ inactivation could indicate that the cells are limited in their activity or better protected, if they are already embedded in gels, when they come into contact with H_2_O_2_. The crosslinking studies also showed that gelation prior to crosslinking requires significantly longer crosslinking times than without gelation. Therefore, it can be concluded that the bioprinted SF10‐ECM5‐G3 samples require significantly longer crosslinking times (109 min) than casted samples (31 min). While the addition of ECM increased the fluorescence maximum, the addition of G decreased it, although G should also provide crosslinking sites having ≈1% tyrosine content.^[^ ^33^ ^]^ Another influence on the crosslinking kinetics besides the presence of cells was exhibited by temperature. Crosslinking SF with HRP and H_2_O_2_ at 37 °C is well explored.^[^ ^16^, ^17^ ^]^ However, less is known about the reaction at 20 °C. Since the free HRP enzyme activity gradually increases between 25 and 45 °C, reactions at lower temperatures were expected to be slower.^[^ ^34^ ^]^ Although the crosslinking times at 20 °C were significantly slower for all conditions subjected to 20 and 37 °C, the higher fluorescence maxima indicated the formation of more crosslinks (Figures 2 and 3).

The production of meniscus implants requires shape and weight fidelity of the used biomaterials to keep the specific meniscus architecture until new tissue is formed. Therefore, the hydrogel height, diameter, and wet weight were monitored for 14 days shown in Figure S5 (Supporting Information). to identify the most suitable biomaterial formulation. Within the SF‐based hydrogels, the height of SF10, SF10‐ECM2.5, and SF10‐ECM5 did not decrease significantly, and they also kept over 90% of their diameter. SF‐based hydrogels were already shown to exhibit slow degradation and to maintain their size and weight over several weeks.^[^ ^17^ ^]^ In contrast, the wet weight of our SF‐based hydrogels reduced significantly to 45–75% of their initial weights. Since hydrogels with a high β‐sheet content were reported to have especially slow degradation, it is not surprising that hydrogel degradation occurred within the first days, where β‐sheets were not as developed as in later stages.^[^ ^35^ ^]^ In contrast to the height and diameter change, the absolute weight loss was not dependent on the SF concentration. The high weight loss for ECM‐supplemented gels between days 1 and 7 was dependent on the ECM concentration, which indicates that ECM is degraded or dissolved faster than SF. Since 7.5% and 10% ECM resulted in a fast reduction of height, diameter, and weight, the usage of 2.5% or 5% ECM appeared more suitable. Nevertheless, the fast solvation or degradation of ECM questioned its usability for promoting cell proliferation and differentiation and suggested that additional gelation or crosslinking mechanisms should be introduced.

Another important feature of hydrogels for meniscus TE is the Young's modulus characterizing the elasticity in tension or axial compression. Human menisci were already reported to have an average axial compressive modulus of 83.4 kPa at 12% strain at equilibrium, although the values differ between the regions of the meniscus.^[^ ^36^ ^]^ Our study averaged samples from three donors and three locations within the menisci and resulted in a similar value of 150 ± 90 kPa (SD) at 10% strain in Figure 5. The Poisson ratio was 0.8 ± 0.4 (SD) and had a high variability too, which was probably related to the anisotropic tissue. This value is higher than a Poisson ratio of 0.5 reported in the literature.^[^ ^36^ ^]^ Properties like the Young's modulus and the Poisson ratio can be engineered within SF hydrogels by varying the crosslinking degree, where increasing crosslink density is associated with stronger and more brittle gels.^[^ ^37^ ^]^ Although higher SF concentrations should facilitate the formation of more crosslinks and stiffer gels, the crosslinking kinetics measurement already revealed that SF7.5 and SF10 probably formed less crosslinks (Figures 2 and 5). This finding correlated with the lower Young's moduli of SF7.5 and SF10 in comparison to SF5. While crosslinking density determines the gel stability in the beginning, the Young's modulus increase over time is mainly dependent on β‐sheet formation. The β‐sheet formation can be guided by crosslinked tyrosine.^[^ ^15^ ^]^ Gels with higher SF concentrations were already reported to result in higher compressive moduli due to self‐assembly of movable hydrophobic chains into crystalline β‐sheet structures.^[^ ^38^ ^]^ While SF2.5 resulted in the lowest Young's modulus as expected due to a lower density of hydrophobic chains, SF5 had a surprisingly high Young's modulus of 1.88 MPa (Figure 5). The higher value in comparison to the Young's modulus of SF7.5 and SF10 gels might be caused by a combination of higher crosslinking density and more β‐sheet structures. The addition of any ECM concentration to SF10 gels reduced the Young's modulus to below 0.23 MPa, which was more similar to the Young's modulus of the human meniscus. The decrease was probably caused by the entanglement of ECM molecules with SF resulting in the hindrance of SF self‐assembly and β‐sheet formation.^[^ ^38^ ^]^

Determination of the Young's modulus indicated that biomaterial maturation in SF10‐ECM5‐G3 gels was still ongoing until day 28 (Figure 7). Studies with synthetic filaments showed that constructs with higher infill densities exhibited greater mechanical strength.^[^ ^39^, ^40^ ^]^ Therefore, our 3D printed cylinders designed to have 0.5 mm initial grid spacing exhibited lower compressive strengths than casted samples. In comparison, higher compressive moduli of ≈188 and 150 kPa and were already obtained for cell‐free printed SF‐ or ECM‐based hydrogels for meniscus TE, where cytotoxic crosslinking mechanisms were applied.^[^ ^14^, ^41^ ^]^ The inclusion of IFP‐MSCs reduced the Young's modulus of our casted samples, which can be attributed to the reduced number of formed crosslinks determined in the crosslinking kinetics experiment (Figures 3 and 7). However, the cell inclusion increased the mean Young's modulus to over 150 kPa for the printed samples. In contrast to the casted samples, a constant value for the printed samples could be explained by the fact that the crosslinking kinetics were not changed with the addition of cells in the gelled samples. Nonetheless, the increase could not be traced back to differences in crosslink number, cell density or extensive β‐sheet formation. It could be at least partially explained by the significantly delayed RC reduction in the printed samples with IFP‐MSCs (Figure 6). We also hypothesized that the printed constructs with IFP‐MSCs were able to resist mechanical deformation better due to aligned cell‐induced gel contraction, which caused diameter reduction by up to 24% and height contraction up to 14% (data not shown).

ATR‐FTIR measurements in Figure 6 gave insights in the secondary structures present in the hydrogels over the course of 28 days. β‐sheet formation occurred more slowly in printed cell‐free controls in 1X PBS than in casted ones. The slower formation was probably caused by lower SF amounts in the constructs due to spacings in the printed grid. Interestingly, the shear applied through 3D printing did not result in more β‐sheets in contrast to recent findings.^[^ ^42^ ^]^ In general, high shear force, certain ion concentrations and low pH could induce increased β‐sheet formation.^[^ ^43^ ^]^ Application of growth medium further delayed β‐sheet formation in the printed cell‐free controls. It contains several components like salts, amino acids, and vitamins that could influence the crosslinking reaction and subsequently the formation of β‐sheets. One component that was already shown to accelerate di‐tyrosine crosslinking of SF gels is phenol red.^[^ ^44^ ^]^ Phenol red can also be oxidated by HRP and H_2_O_2_, which facilitates crosslinking with tyrosyl radicals. Therefore, crosslinking phenol red or amino acids contained in the growth medium could have reduced the number of SF di‐tyrosine crosslinks and the correlating β‐sheets. The effect of growth medium on printed samples could have been stronger than on casted samples, because growth medium with H_2_O_2_ was applied as a bath for crosslinking initiation after 3D printing, while growth medium was only added to casted samples after 30 min of crosslinking. When IFP‐MSCs were added into casted and printed gels cultured in growth medium, the peak intensities reduced substantially, which could be due to the detoxification of H_2_O_2_ by cells in the casted samples. β‐sheet peak intensity decreased in all samples in differentiation medium between day 14 and day 28, which indicated remodeling processes.

Casting and printing the hydrogels with IFP‐MSCs resulted in low cell viabilities of 59% and 56% on day 0 shown in Figure 8. The low viability of the casted samples was attributed to the crosslinking mechanism with the exposure to potentially cytotoxic H_2_O_2_. H_2_O_2_ is known to induce apoptotic cell death even at low (0.1–10 mm) concentrations, but its participation in the crosslinking reaction should minimize its cytotoxicity.^[^ ^38^ ^]^ Nevertheless, crosslinking of the casted gels was shown to be reduced by the presence of IFP‐MSCs in Figure 3, wherefore more residual H_2_O_2_ could have harmed the cells. As crosslinking of the previously gelled samples was not affected by the presence of cells, H_2_O_2_ might not have harmed the IFP‐MSCs in the printed samples. 3D bioprinting is already known to impact cell viability because of high shear stress. For example, Osama et al. used sonication to initiate β‐sheet formation allowing SF (2–4% w/v) gelation and evaluated the effect of shear stress on mouse MSC viability.^[^ ^45^ ^]^ The liquid form or the post‐gelled form were extruded through different needle gauges (21, 25, and 30G), where cell viability was significantly lowered by extrusion of 4% gelled SF.^[^ ^45^ ^]^ This indicates that our use of a needle with 27G and 10% SF already harmed the cells. Further studies on cell viability in printed but non‐crosslinked gels and with larger needle diameters would be required to determine, if the low viability was caused by crosslinking, printing, or the high concentrations of SF and ECM. 2 h were chosen as general crosslinking time, since the crosslinking time of previously gelled SF10‐ECM5‐G3 with IFP‐MSCs was 109 min at 20 °C. However, the time could have been reduced for the casted samples, since their crosslinking was done in 31 min. Reduced exposure time and washing steps could have improved the cell viability in the casted gels.

In general, the cell viabilities increased to over 84% during further cultivation in growth and differentiation medium. The living cell number approximately doubled until day 14 for all conditions and changed only slightly in the casted samples until day 28. In comparison, 3D printing of SF‐G hydrogels with subsequent seeding of porcine meniscal fibrochondrocytes was already shown to facilitate proliferation by 1.8–2 times within 21 days.^[^ ^14^ ^]^ However, in contrast to the casted samples the cell numbers in our printed samples dropped below the initial cell number. Although 3D bioprinting did not affect the initial cell number and viability in comparison to the casted samples, it could impact the cells due to the stiffer environment or the construct architecture itself. Scaffolds with pore sizes of ≈300 µm were already reported to stimulate higher sGAG production and SOX9 and COL2 gene expression than scaffolds with smaller pore sizes, while COL1 gene expression was lower.^[^ ^46^ ^]^ Despite the apparent larger pore size of printed cell‐free controls in comparison to casted ones, there may be variations in pore size among samples containing IFP‐MSCs, since cells were identified to influence crosslinking kinetics, secondary structures, and Young's modulus. SEM images of printed samples with IFP‐MSCs would give insights into any alterations in pore size and distribution. The increased stiffness in the printed samples with IFP‐MSCs could limit proliferation. On the one hand, 12% SF‐only hydrogels with an equilibrium modulus of 441 kPa were already found to limit BMSC proliferation in comparison to 4% SF hydrogels with 63 kPa, which was justified by mass transport limitations and mechanical restrictions.^[^ ^47^ ^]^ On the other hand, bioprinting through a nozzle with a diameter of 250 µm did not lead to any harmful effect on human MSCs, when they were encapsulated in bioinks with 8% SF and 15% G.^[^ ^48^ ^]^ Therefore, we hypothesized that our limited viability and proliferation in the printed samples was caused by a combination of shear forces, crosslinking agents and crosslink density, material stiffness, and higher ECM or G loss than in the casted samples.

SF was already shown to increase expression of COL2, ACAN, and SOX9 in rat MSCs with chondrogenic medium by Zheng et al. and was even described to upregulate SOX9 and ACAN expression in canine MSCs without chondrogenic supplements by Voga et al.^[^ ^49^, ^50^ ^]^ Collagen and ECM have been demonstrated to possess such properties as well.^[^ ^7^, ^51^, ^52^ ^]^ Intrinsic potential of hydrogels with SF or ECM to upregulate chondrogenic markers could not be confirmed in this study, since only COL2A1 was significantly upregulated in casted and printed hydrogels in growth medium, when compared to the 2D control in growth medium (Figure 9). The application of chondrogenic differentiation medium resulted in a significant upregulation of COL1A1, COL2A1, ACAN, and SOX9 expression in casted gels relative to those cultured in growth medium. Expression of those genes was also significantly increased in casted samples in comparison to 2D controls, except for COL1A1 on day 14. The promotion of chondrogenic differentiation by MSCs inclusion in SF‐based hydrogels was in accordance with findings in the literature. For example, Zuluaga‐Vélez et al. showed that inclusion of human MSCs in 4% SF hydrogels supplemented with TGF‐β3 significantly increased ACAN and COL2A1 expression in comparison to pellet culture.^[^ ^53^ ^]^ In contrast to the casted hydrogels, the IFP‐MSCs in the printed samples showed limited chondrogenic differentiation potential, where COL2A1 and SOX9 were only significantly upregulated on day 28 in differentiation medium compared to growth medium (Figure 9). These findings stand in contrast with previous studies in the literature, where 3D bioprinting with SF‐based bioinks did not hinder chondrogenic differentiation. For example, Das et al. found that human MSCs cultured in chondrogenic differentiation medium exhibited higher expression of SOX9, COL2, and ACAN, when they were bioprinted in enzymatically crosslinked bioinks with 8% SF and 15% G than in alginate bioinks.^[^ ^48^ ^]^ In another study, 3D bioprinting was even found to induce upregulation of chondrogenic markers without exogenous differentiation signals. Yan et al. used 10% SF and 10% hydroxypropyl cellulose methacrylate (HPCMA) bioinks with rabbit MSCs to investigate repair of cartilage defects.^[^ ^54^ ^]^ SOX9, ACAN, and COL2A1 were significantly upregulated in SF‐HPCMA scaffolds in comparison to SF scaffolds.^[^ ^54^ ^]^

As already described for the cell number decrease, the limited chondrogenic differentiation in the printed samples may be attributed to the elevated mean compressive Young's modulus of 0.19 MPa on day 28 (Figure 7). However, this hypothesis is contradicted by the fact that differentiation was not impaired by high compressive moduli of 0.402 MPa in the study of Yan et al.^[^ ^54^ ^]^ Decreasing the SF concentration and reducing shear stress during 3D bioprinting might enhance initial cell viability and consequently chondrogenic differentiation. Reducing the concentration might not even lead to a decreased Young's modulus in the end, since SF5 had a significantly higher Young's modulus than SF10 in the concentration determination experiments (Figure 5). Furthermore, Onofrillo et al. found that 6% gelatine methacryloyl (GelMA) led to significantly higher expression of SOX9 on day 28 in comparison to 10% GelMA.^[^ ^55^ ^]^ Intriguingly, chondrogenesis within 6% GelMA hydrogels resulted in a significant increase in compressive modulus, elevating it from the lowest on day 1 to the highest on day 28 among the 6%, 8%, and 10% GelMA hydrogel groups.^[^ ^55^ ^]^ This suggests that mechanical properties could be increased by chondrogenesis. Another approach to increase cell proliferation and differentiation could be to use two inks for scaffold preparation similar to the approach of Chae et al.^[^ ^8^ ^]^ They aimed at 3D bioprinting a meniscus using one ink with polyurethane and polycaprolactone and one bioink with human MSC‐laden porcine meniscus‐derived extracellular matrix.^[^ ^8^ ^]^ One ink could contain high SF concentrations with G and one bioink could embed IFP‐MSCs in the combination of ECM and G. Keeping in mind that cylinders with grid infills might not be the best strategy for chondrogenesis, future studies could also investigate the contribution of printing the bioink in the architecture of the natural meniscus to enhance chondrogenic differentiation in bioprinted scaffolds.

Despite the encouraging cytocompatibility and chondrogenic differentiation capacity of IFP‐MSCs in the casted samples and compressive strength exhibited by the 3D bioprinted samples, further characterization is required to elucidate their mechanical properties, including shear modulus and tensile strength. The declining cell number in the printed samples was contrary to our expectations and should also be addressed in the following study by closely investigating each parameter including shear stress, presence of crosslinking agents, and ECM‐G contribution. Achieving ECM gelation by decellularization optimization and adapting printing parameters could greatly increase cell viability and differentiation capacity. Furthermore, the application of hydrostatic stimulation could increase the number of cells, ensure matrix production, and provide a more physiologically relevant environment. Another valuable tool to improve scaffold functionality for mimicry of the meniscus is the 3D printing of the specific collagen architecture. Our investigation into 3D bioprintable hydrogels with high concentrations of SF and ECM provided a foundation for future research aimed at fabricating such a meniscus implant. It has shown how the presence of cells can affect crosslinking and consequently the secondary structure, the mechanical properties, cell viability, and chondrogenic differentiation capacity in casted and printed hydrogels.

## 4 Conclusion

SF‐ECM‐G was selected as the most promising composite hydrogel for 3D‐bioprinting due to its excellent printability and fast crosslinking when subjected to HRP and H_2_O_2_. The resulting hydrogels with high water contents and good shape and weight retention exhibited compressive Young's moduli comparable to human OA menisci, suggesting their suitability for meniscus TE. IFP‐MSCs were successfully included in casted and printed hydrogels. While cells were able to proliferate in the casted samples despite an initial low cell viability and demonstrated significant upregulation of COL1A1, COL2A1, ACAN, and SOX9 expression upon stimulation with chondrogenic medium, proliferation, and differentiation were hindered in the printed samples. A comparison of 3D bioprinting and casting also revealed differences in metabolic activity, secondary structure, and Young's modulus. IFP‐MSCs were found to affect crosslinking kinetics, secondary structures, and mechanical strength. Overall, the findings presented in this study highlight the promising potential of SF‐ECM‐G hydrogels for future studies on meniscus implants.

## 5 Experimental Section

### Preparation of SF Solution

*Bombyx mori* silk cocoons (Seidentraum, Germany) were processed into SF solution according to published protocols.^[^ ^56^ ^]^ Briefly, 5 g of cut cocoons were degummed in 2 L boiling 0.02 m Na_2_CO_3_ (Sigma–Aldrich, Austria) for 60 min. The SF fibers were washed in ultrapure water (UPW). Then, the fibers were spread and dried at RT for 48 h, before they were dissolved in 9.3 mM LiBr (Sigma‐Aldrich, Austria) at a concentration of 20% (w/v) for 3 h at 60 °C. The solution was transferred into a Spectra/Por dialysis membrane (Carl Roth, Germany) with a molecular weight cutoff of 3.5 kD. It was dialyzed against UPW for 3 days with 6 water changes in total. Afterward, the membrane with SF solution inside was stored at 4 °C for at least 7 days to remove some water, before the solution was centrifuged at 4600 rpm for 10 min. The supernatant concentration was determined by drying for 12 h at 90 °C. The mean of three samples was used as the final concentration of the SF solution, which was stored at 4 °C until use.

### Preparation of ECM Powder

Bovine menisci were obtained from the slaughterhouse and processed into ECM powder by decellularization and digestion by the adaption of published procedures.^[^ ^57^, ^58^ ^]^ Concisely, the menisci were cut, frozen, lyophilized for 72 h, and cryomilled. 1 g of ground tissue was agitated in 40 mL of 1X phosphate‐buffered saline (PBS, Thermo Fisher Scientific, Austria) containing 1% Triton X‐100 (Sigma‐Aldrich, Austria) at 4 °C for three days. Then, the tissue was washed three times in 1X PBS at 4 °C for 30 min each, before it was transferred to 40 mL of RNA‐DNA enzyme extraction buffer. This buffer containing 2.5 kU benzonase (Sigma‐Aldrich, Austria) and 8 mm MgCl_2_ (Sigma–Aldrich, Austria) in UPW was adjusted to pH 8 using 1M NH_4_OH. The tissue was continuously agitated at 37 °C for 24 h prior to the final six washes in 1X PBS. The decellularized tissue was frozen, lyophilized, and cryomilled again. The ECM powder was then digested at a concentration of 5% (w/v) in 0.1 m HCl with 0.5 mg mL^−1^ porcine pepsin (Sigma–Aldrich, Austria) for 2 h at 37 °C. The solution was neutralized with 0.1 N NaOH and 10X PBS. It was frozen, lyophilized, and stored at 4 °C until use.

To confirm the complete decellularization and to determine the influence of the process on ECM components, assays were applied to bovine meniscus samples and the resulting ECM powder. For the DNA and sGAGs content, 150 mg were resuspended in 1 mL 1X PBS, supplemented with 40 µL proteinase K (50 µg mL^−1^) and incubated at 56 °C overnight. Proteinase K was inactivated by incubation at 90 °C for 10 min, before the samples were centrifuged for 5 min at 12 300 rpm. The DNA content was measured with the kit “Quant‐iT PicoGreen dsDNA Assay Kits and dsDNA Reagents” (Thermo Fisher Scientific, Austria). The sGAG content determination was conducted according to published protocols.^[^ ^59^ ^]^ Briefly, 1 mL 1,9‐dimethyl‐methylene blue (DMMB, Sigma‐Aldrich, Austria) was mixed with 100 µL sample. The mix was centrifuged for 10 min at 12 000 × g and the pellet was dissolved in a decomplexation solution. After 30 min incubation on a shaker, the absorbance at 656 nm was measured photometrically (Amersham Bioscience plc, Amersham, UK). The sGAG content was determined by the use of a standard curve with shark chondroitin sulfate (Sigma–Aldrich, Austria). The “Hydroxyproline Assay Kit” (Sigma–Aldrich, Austria) was used to measure the collagen content. All assays were performed with triplicates and according to the manufacturer's protocol.

### Hydrogel Preparation for Concentration Establishment

Crosslinking with 10 u mL^−1^ horseradish peroxidase (HRP, Sigma‐Aldrich, Austria) and 0.01% H_2_O_2_ (Sigma‐Aldrich, Austria) was chosen as the most promising mechanism to obtain stable SF‐based hydrogels.^[^ ^17^ ^]^ To establish the ideal concentrations of SF and ECM, hydrogels composed of the single and mixed components were prepared for the crosslinking kinetics experiment, wet weight and size measurement, and unconfined compression tests. For the conditions that only contained SF (10%, 7.5%, 5%, and 2.5% (w/v)), a 10% SF solution was diluted accordingly. For the ones that only contained ECM, the lyophilized ECM powder was dissolved in sterile water at 37 °C. The conditions with both components were prepared by dissolving the appropriate amount of ECM powder directly in a 10% SF solution at 37 °C. 10% SF with 10%, 7.5%, 5%, and 2.5% ECM were chosen for the composites, since 10% SF was expected to yield the most stable hydrogels.

The preparation procedure for the crosslinking kinetics experiment is described in the section “Fluorescence spectroscopy”. For the other measurements, 10 u mL^−1^ HRP and 0.01% H_2_O_2_ were added to each solution, before 4.8 mL were cast into one well of a six‐well plate. The solutions were allowed to gel at 37 °C for 2 h and cylinders with a diameter of 8 mm were punched out with a biopsy punch (Henry Schein, Austria). Each sample was stored in 1 mL 1X PBS at 37 °C (or 4 °C in case of the samples containing only ECM) until further use with a PBS change once a week. Three replicates were prepared for each day and each condition. On the days 1, 7, and 14 the analyses, which are described in the section “Wet weight and size measurement and Unconfined compression” were performed.

### Fluorescence Spectroscopy

The formation of di‐tyrosine crosslinks was monitored by fluorescence spectroscopy.^[^ ^38^, ^60^ ^]^ All components except for H_2_O_2_ were mixed and each solution was prepared to fill three wells of a 96‐well plate with 100 µL per well. Then, crosslinking was induced by the addition of 10 u mL^−1^ HRP and 0.01% H_2_O_2_. The crosslinking kinetics were monitored at 37 °C for the biomaterial characterization and at 20 °C for cell inclusion by measuring fluorescence emission at 460 nm after excitation at 360 nm using a plate reader (BioTek Synergy 2, Agilent, United States). The measurement was done every 5 min for 180 min and the samples were normalized to the first measurement. If there was a clear plateau, the crosslinking point was defined as the time at which the slope of the fluorescence was less than or equal to 1 for the first time.

### Wet Weight and Size Measurement

Triplicates of each casted gel were weighed on day 1, 7, and 14. Additionally, pictures were taken to record the course of transparency change caused by β‐sheet formation, and the diameter and the height were measured three times on three gels with a sliding gauge.^[^ ^26^ ^]^

### Unconfined Compression

For the unconfined compression tests, the samples were placed onto the metal plate of the MFT‐5000 Multi‐Function Tribometer (Rtec instruments, United States) and the stamp was lowered, until it touched the sample. 1 N was applied to the samples within 2 min with a linear force increase. Then, the diameter was measured three times with a sliding gauge. The applied stress *F_z_* [N] was converted to the stress *σ* [MPa] with the surface area of the sample *a* [m^2^]. The axial strain *ε* [‐] and the lateral strain were calculated. Then, the Young's modulus [MPa] was calculated by dividing the stress *σ* [MPa] by the axial strain *ε* [‐]. The calculated Young's modulus was also plotted over time. The mean of the last 10 values of the Young's modulus before an axial strain of 10% was taken to specify one Young's modulus value. Finally, the Poisson's ratio *ν* [−] was calculated by dividing the lateral strain [−] by the axial strain *ε* [−].

### Human Meniscus Sampling

To determine a target value for the Young's modulus and the Poisson ratio of the hydrogels, human meniscus samples were received from the University Hospital Krems from three OA patients who underwent total knee arthroplasty (TKA). Written informed consent was obtained from all donors. Ethical approval for the use of human menisci samples was obtained from the Ethics Committee of Lower Austria (GS4‐EK‐4/763‐2021). Three cylindrical samples with a diameter of 8 mm were punched out of each meniscus. The diameter and the height of each sample were measured three times with a sliding gauge and the unconfined compression tests proceeded as described in the section “Unconfined compression.”

### 3D‐Printing of Hydrogel Formulations

The combination of 10% SF and 5% ECM with 10 u/mL HRP and 0.01% H_2_O_2_ was used for extrusion‐based 3D‐printing with an Allevi 3 bioprinter (Allevi, United States). 3% porcine skin gelatin type A (G, Sigma–Aldrich, Austria) were added to the mixture to induce gelation at 20 °C. By this strategy, HRP could be included in the soft hydrogel for 3D‐printing and H_2_O_2_ could be provided after the print in the form of a H_2_O_2_ bath. The printability of SF10‐ECM5‐G3 at 20 °C was compared to a combination without G at 4 °C. 10% SF were supplemented with 10% ECM, since this ECM concentration was sufficient to induce strong gelation at 4 °C to obtain printable hydrogels. Both gelled mixtures (SF10‐ECM5‐G3 and SF10‐ECM10) contained 10 u mL^−1^ HRP and were extruded with a 27G (210 µm inner diameter) sized nozzle. Cylindrical discs with a diameter of 8 mm and grid infill were extruded with different grid spacings (0.3, 0.4, 0.5, 0.75, and 1 mm). The discs were photographed before 0.5 mm spacing was chosen for printing full cylinders with a height of 5 mm. Pictures of the cylinders were taken after 5, 10, and 17 layers. No H_2_O_2_ bath was added for this printability test, wherefore no crosslinking was induced after the print.

### IFP‐MSC Preparation

According to published protocols IFP‐MSCs were obtained from three OA patients undergoing TKA.^[^ ^19^, ^61^ ^]^ Written informed consent and the ethical approval was obtained (GS4‐EK‐4/763‐2021). Briefly, IFP tissue was minced and digested with 9000 units of collagenase I (Sigma–Aldrich, Austria) per 10 g of tissue in 15 ml Gibco DMEM (Thermo Fisher Scientific, Austria) for 2 h at 37 °C. Afterward, the suspension was filtered through a 40 µm cell strainer (Thermo Fisher Scientific, Austria), cells were washed in 1X PBS and resuspended in MSC growth medium (Gibco DMEM with high glucose, GlutaMAX and pyruvate (Thermo Fisher Scientific, Austria), 10% FCS, 1% nonessential amino acids (both from Thermo Fisher Scientific, Austria), 2% Penicillin/Streptomycin, 1% Amphotericin B, and 1 ng mL^−1^ Fibroblast Growth Factor‐Basic (all from Sigma–Aldrich, Austria)). Cells were expanded at 37 °C with 5% CO_2_ until 80–90% confluency with a medium change every 2–3 days, detached with accutase, centrifuged, and diluted in freezing media (10% DMSO (Sigma–Aldrich, Austria) and 90% FCS). 10^6^ cells in 1 mL of freezing media were stored in cell freezing containers in a −80 °C freezer overnight. The next day samples were transferred to the liquid nitrogen tank. Consequentially, cells from passage 2 to passage 8 were used for the experiments. For thawing the cells, the samples were immediately transferred from liquid nitrogen to the 37 °C water bath for 2 min. Thawed samples were diluted using prewarmed MSC growth medium and centrifuged. Then the cells were seeded in MSC growth medium and incubated at 37 °C with 5% CO_2_.

### 3D‐Bioprinting and Casting with IFP‐MSCs

The SF10‐ECM5‐G3 solution was prepared as described in 5.8 and 10 u mL^−1^ HRP were added, before 10^6^ IFP‐MSCs/mL were resuspended after centrifugation. For the casted cylinders, 0.01% H_2_O_2_ was added, and 4.8 mL were transferred into each well of six‐well plates. The solutions were allowed to gel at 37 °C for 2 h, although MSC growth medium was already added on top after 30 min. Cylinders with a height of 5 mm were punched out with a biopsy punch with a diameter of 8 mm and each sample was stored in 1 mL MSC growth medium at 37 °C. For the printed cylinders, the solution without H_2_O_2_ was aspirated into a syringe, which was transferred to the 3D‐bioprinter. The solution was allowed to gel for 30 min at 20 °C, before cylinders with a height of 5 mm, a diameter of 8 mm and a grid spacing of 0.5 mm were extruded through a 27G sized nozzle with 6 mm s^−1^ and ≈30 PSI into wells of a 24‐well plate. 1 mL of MSC growth medium with 0.05% H_2_O_2_ was added to each well to provide 0.01% H_2_O_2_ to each cylinder. The cylinders were incubated at 20 °C for 2 h, before the medium was refreshed and the plate was transferred to 37 °C. Starting on day 3, half of the samples were incubated in chondrogenic differentiation medium (Gibco DMEM with high glucose, GlutaMAX and pyruvate (Thermo Fisher Scientific, Austria), 1% ITS, 100 nm dexamethasone, 50 µg mL^−1^ ascorbic acid, 2% Penicillin/Streptomycin, 1% Amphotericin B (all from Sigma–Aldrich, Austria), 10% FCS, 1% nonessential amino acids (both from Thermo Fisher Scientific, Austria) and 5 ng mL^−1^ TGFβ‐3 (PeproTech, USA)). The medium was changed twice a week for both conditions (MSC growth and chondrogenic differentiation medium).

### Metabolic Activity of IFP‐MSCs

A vertical slice of the middle (see Figure S1, Supporting Information) of the casted and printed cylinders was cut on day 0 and day 28 for confocal microscopy. The remaining two halves of each gel with the IFP‐MSCs of one donor were minced, weighed, and investigated via the XTT assay (Roche Diagnostics, Germany) according to the manufacturer's protocol. The samples were agitated for 4 h in the assay reagents at 37 °C. Then, the supernatant was discarded, and the colored gel pieces were agitated for 1 h in DMSO at 37 °C. The supernatant was used to measure the absorbance at 492 and 690 nm.

### ATR‐FTIR Spectroscopy

The hydrogels were washed in D_2_O (Carl Roth, Germany) three times for 30 min, before they were placed on the ATR‐FTIR device (Perkin Elmer, Austria). Four scans with a resolution of 1 cm^−1^ between 450 and 4000 cm^−1^ were averaged. Since the region of interest for SF secondary structures ranges from a wavenumber of 1600 to 1700 cm^−1^ (amide I region), the peak of this section was used for peak deconvolution in OriginPro 2024. The option “FitPeaks” was chosen in the Peak Analyzer Tool with baseline subtraction. Hidden positive peaks were found by calculating the residual after the first derivative, which resulted in 2–6 peaks with fixed centers. The wavenumber at each peak maximum was used to determine the type of secondary structure. Peaks with a maximum at a wavenumber larger than 1616 cm^−1^ and smaller than 1638 cm^−1^ were assigned to β‐sheets, while peaks with a maximum at a wavenumber larger than 1639 cm^−1^ and smaller than 1656 cm^−1^ were assigned to random coils (RCs). The ratios of β‐sheets to total amide I and RCs to total amide I were calculated with the individual peak areas.

### Viability Assay with Confocal Microscopy

Vertical hydrogel slices of the gel middle (see Figure S1, Supporting Information) were washed in 1X PBS three times, before they were incubated in 1 mL 1X PBS with 4 µm calcein AM (Thermo Fisher Scientific, Austria) and 8 µm EthD‐1 ethidium homodimer‐1 (EthD‐1) (Thermo Fisher Scientific, Austria) for 1 h at 37 °C. Then, the gels were washed again and visualized with the 10X objective of the TCS‐SP multi‐Photon confocal microscope (Leica, United States). Images were taken of the top, the bottom, the side, and the middle of each vertical section.

### SEM

The samples that were used for the wet weight measurement were frozen and dried for at least 48 h in a freeze dryer (Zirbus, Germany). The dry samples were weighed to calculate the water content by dividing the weight difference by the wet weight, before a vertical slice was cut out via a scalpel to view the inside of the freeze‐dried sample. The pieces were placed on a sample holder and sputter coated with a gold film using a Rotary Pumped Coater (Quorum, United Kingdom) prior to imaging with a scanning electron microscope (Hitachi, Japan).

### RNA Extraction and DNA Digestion

One cylinder with IFP‐MSCs per condition was minced and put in a MagNA Lyser tube (Roche Diagnostics GmbH, Germany) with 500 µL TRIzol Reagent (Thermo Fisher Scientific, Austria). 2D controls, where 19 000 IFP‐MSCs were seeded into each well of a 24‐well plate on day 0 and also incubated with growth medium or chondrogenic differentiation medium until day 14 and day 28, were used as a reference. 500 µL TRIzol Reagent was used to suspend the cells from the 2D controls and also transfer them into MagNA Lyser tubes. All samples were stored in liquid nitrogen. After thawing for 2 min, 100 µL chloroform (Thermo Fisher Scientific, Austria) were added to each sample. Then, the samples were transferred to the MagNA Lyser (Roche Diagnostics GmbH, Germany) and subjected to one run at 6500 rpm for 20s. The TRIzol RNA extraction was proceeded as outlined in the manufacturer's manual. Subsequently, DNA digestion was performed with DNase I (Thermo Fisher Scientific, Austria) according to the manufacturer's instructions. The RNA was stored at −80 °C until cDNA synthesis and quantitative real‐time polymerase chain reaction (qRT‐PCR).

### cDNA Synthesis and qRT‐PCR

RNA from bacteriophage MS2 (Sigma–Aldrich, Austria) was used to stabilize the isolated RNA during cDNA synthesis with the Transcriptor First Strand cDNA Synthesis Kit (Roche Diagnostics GmbH, Germany) according to the manufacturer's protocol. RT‐qPCR was done using the FastStart Essential DNA Probes Master Kit (Roche Diagnostics GmbH, Germany) following the manufacturer's guidelines. 1 µL cDNA, FastStart Probe Master 2x, hydrolysis probe (final concentration 250 nc), and primers (final concentration of 900 nm) were used for PCR amplification on a Roche LightCycler 96. Collagen type 1 (COL1A1), collagen type 2 (COL2A1), SRY‐box transcription factor 9 (SOX9), matrix metalloproteinase‐3 (MMP3), matrix metalloproteinase‐13 (MMP13), and aggrecan (ACAN) were analyzed using glyceraldehyde‐3‐phosphate dehydrogenase (GAPDH) as reference gene. The 2^−ΔΔCt^ method was used to calculate the fold change gene expression in comparison to 2D controls in growth medium on day 14. The primers for the genes were applied as they have already been published.^[^ ^19^ ^]^

### Statistical Analysis

The statistical calculations were performed using GraphPad Prism version 9.3.1 (Inc., San Diego, USA). Data are given as mean ± standard deviation (SD). Normal distribution was tested using the Shapiro‐Wilk test and Student's *t*‐test was used to compare means between two groups. For comparison between more than two groups one‐way ANOVA with Tukey multiple comparison was applied. For comparisons between more than two groups, where each group consisted of more subunits (like time points) two‐way ANOVA with Tukey multiple comparison was applied. In all cases, significance was attributed when *p* < 0.05 (*), *p* < 0.01 (**), *p* < 0.001 (***) and *p* < 0.0001 (****). The Compact Letter Display was used in diagrams, if too many samples were significantly different to be displayed properly. Different letters above the bars (a–j) indicate statistically significant differences between the groups (*p* < 0.05). Groups sharing a letter are not significantly different (*p* ≥ 0.05).

## Conflict of Interest

The authors declare no conflict of interest.

## Supporting information



Supporting Information

## Data Availability

The data that support the findings of this study are available from the corresponding author upon reasonable request.
