# Supplementary material for: Silk Fibroin‐Based Hydrogels Supplemented with Decellularized Extracellular Matrix and Gelatin Facilitate 3D Bioprinting for Meniscus Tissue Engineering
Source: Macromol Biosci. 2025 Mar 6;25(6):2400515. doi: 10.1002/mabi.202400515 (PMC12169504; doi:10.1002/mabi.202400515)
Supplement: Supplementary file 1 — Supporting Information [file MABI-25-2400515-s001.docx]

# Supplementary Materials


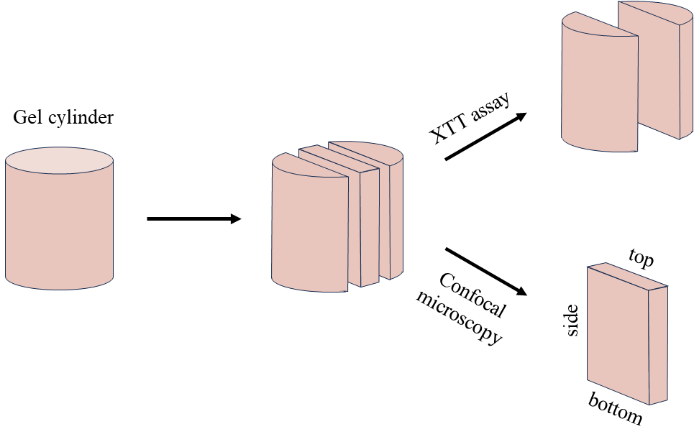


**Figure S 1.** Hydrogel preparation for XTT assay and confocal microscopy.

## Crosslinking kinetics

Due to the results of the ECM gelation measurements, another crosslinking experiment was set up to observe the influence of different H_2_O_2_ concentrations on ECM. Solutions with 10%, 7.5%, 5% and 2.5% (w/v) ECM and 10 u/mL HRP were supplemented with 0%, 0.01%, 0.05% and 0.1% H_2_O_2_. The fluorescence measurement was carried out as described in 5.4.

The lowest measured fluorescence value was subtracted from all other values to indicate the fluorescence drop. Therefore, similar fluorescence starting values of the same ECM concentrations had different starting values. The incubation with 0% H_2_O_2_ led to a slow decrease of fluorescence over 180 min (Figure S 2A), while the application of 0.01% H_2_O_2_ caused a steep decline of fluorescence within the first 15 min. Afterwards, the fluorescence increased again and exceeded the starting values (Figure S 2B). Similarly, 0.05% H_2_O_2_ induced an even steeper fluorescence drop within the first 30 min but failed to reach the initial values within the following rise (Figure S 2C). 0.1% H_2_O_2_ caused the highest drop and failed to induce any fluorescence increase within 180 min (Figure S 1D). Figure S 3 illustrates that the application of different H_2_O_2_ concentrations without HRP only led to small differences in the fluorescence values. Therefore, it can be concluded that only the combination of HRP and H_2_O_2_ induced an extensive fluorescence drop.


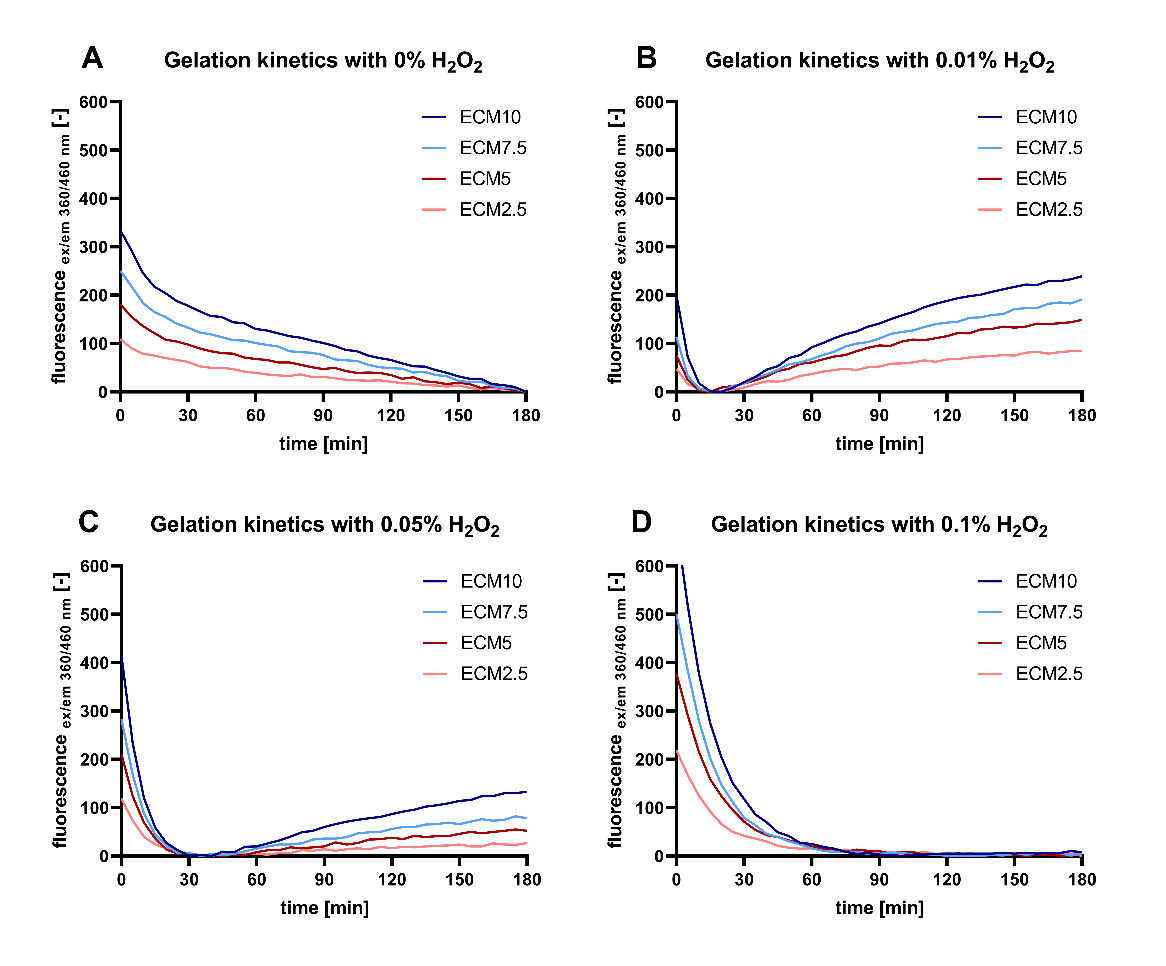


**Figure S 2.** A-D) Crosslinking kinetics of ECM gels with varying H_2_O_2_ concentrations with 10 u/mL HRP at 37°C. (n = 1, with three technical replicates)


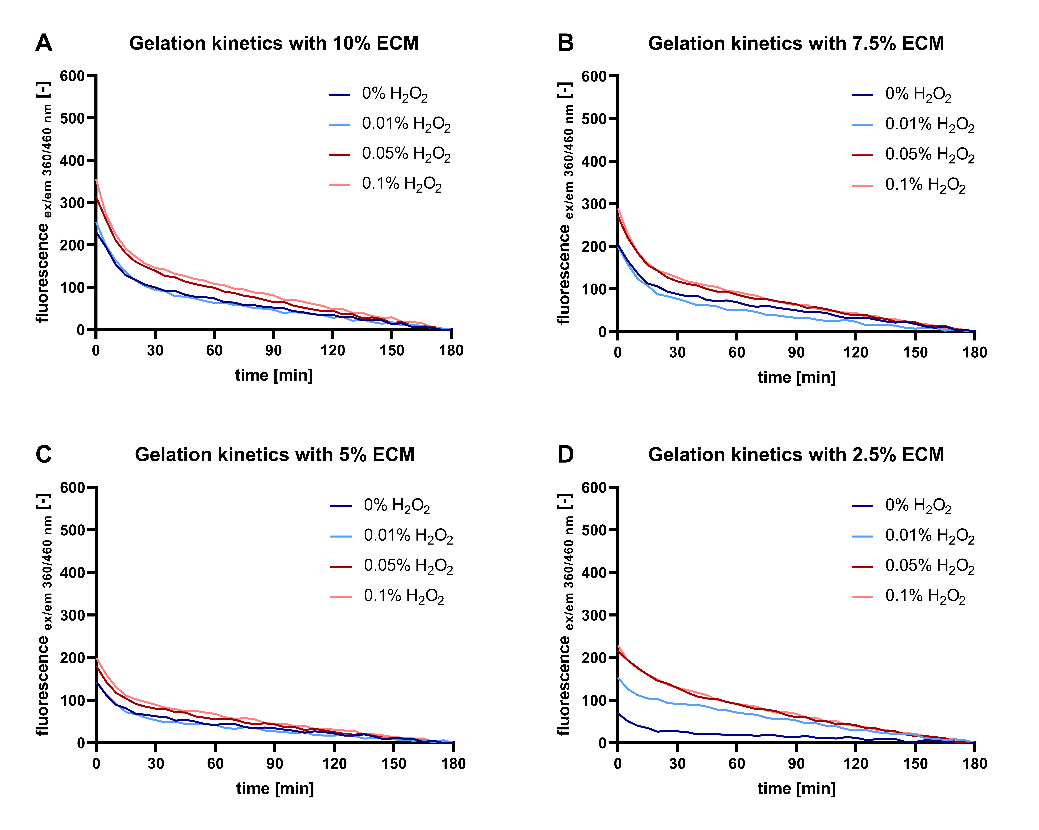


**Figure S 3.** A-D) Crosslinking kinetics of ECM hydrogels with varying H_2_O_2_ concentrations without HRP at 37°C. (n = 1, with three technical replicates)

## Metabolic activity of hydrogel embedded human OA chondrocytes

The biocompatibility of SF and ECM was initially tested with human OA chondrocytes and compared to the one of G. Human articular cartilage was received from the University Hospital Krems from OA patients by informed consent and ethical approval (GS4-EK-4/763-2021). Chondrocyte isolation and expansion were proceeded according to published protocols.^[1]^ Briefly, the cartilage was minced and digested with liberase (Roche Diagnostics GmbH, Germany). The chondrocytes were expanded in chondrocyte growth medium (GIBCO® DMEM/F-12 GlutaMAX™, Invitrogen, LifeTech, Austria) with antibiotics (penicillin 200 U/ml; streptomycin 0.2 mg/ml and Amphotericin B 2.5 μg/ml (Sigma-Aldrich, Germany)), 10% fetal calf serum (FCS, PAA Laboratories, Austria) and 0.05 mg/ml ascorbic acid (Sigma-Aldrich, Germany)) at 37°C with 5% CO_2_ until 80% confluency. Then, cells were harvested with accutase (PAA Laboratories, Austria), counted and seeded as 2D controls and in hydrogels in 96-well plates. All wells contained 3200 cells and 100 µL chondrocyte growth medium, where the 3D cultures were prepared with 100 µL SF-based solution with 10 u/mL HRP, before 0.01% H_2_O_2_ induced crosslinking. The tested conditions included 10% SF supplemented with 5% or 10% of the collagen source (ECM or G) and 10% SF supplemented with 5% ECM and 5% G. The plates were incubated at 37°C with 5% CO_2_ and the medium was changed twice a week. On day 1, 7 and 14, the metabolic activity of the OA chondrocytes was investigated by the XTT assay (Roche Diagnostics, Germany) according to the manufacturer’s protocol. The absorbance at 492 nm and 690 nm was measured by using a plate reader after incubating the samples for 5 h in the assay reagents at 37°C.

Since chondrocytes belong to the most abundant cell types in the human meniscus^[2]^, the biocompatibility of the bioink materials and G as an alternative collagen source and gelling agent was initially tested with human OA chondrocytes from three donors using the XTT assay. Only the metabolic activity of the chondrocytes in 2D culture significantly increased from day 1 to day 7 and day 14. While on day 1 the metabolic activity of all samples did not differ substantially, the metabolic activity in the 2D cell-free control was significantly higher than in the other conditions on day 14. Among the hydrogel samples, SF10 led to the lowest metabolic activity, whereas SF10-ECM5-G5 favored highest metabolic activity, however, without statistical significance compared to day 1 (Figure S 4).


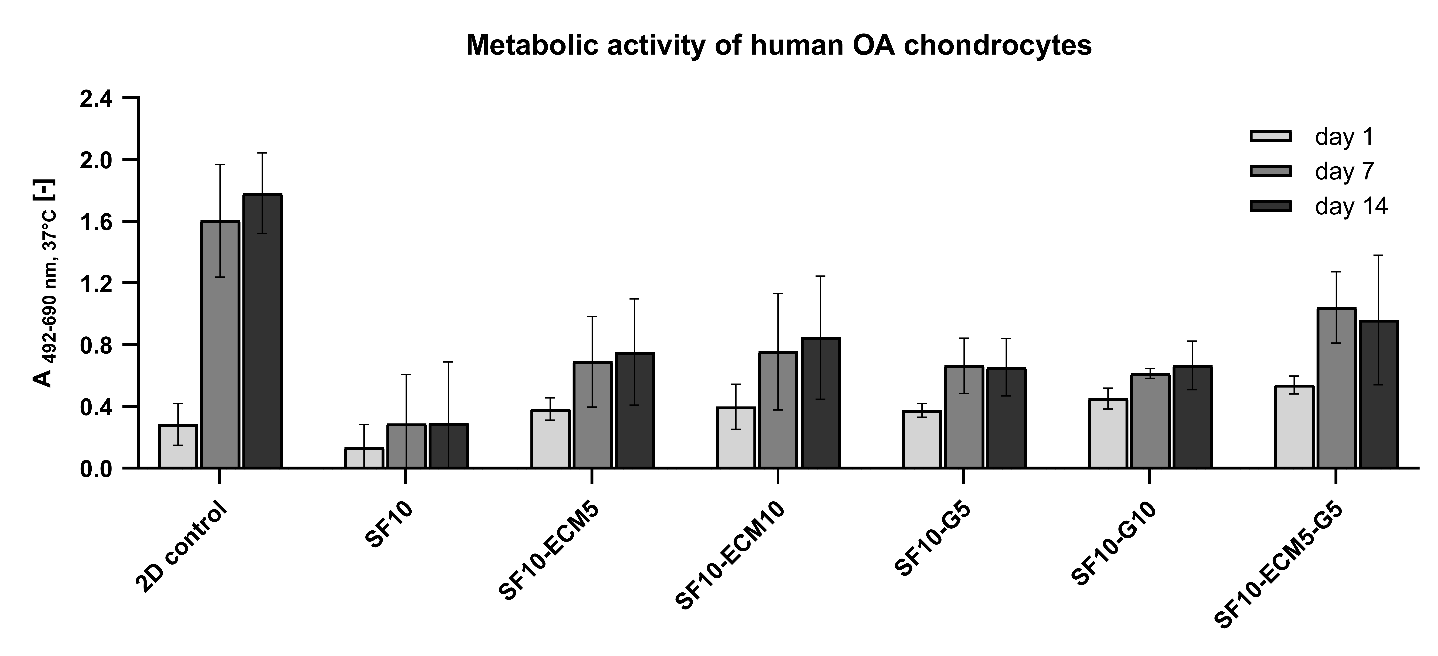


**Figure S 4.** Metabolic activity measured on day 1, 7 and 14 by the XTT assay of human OA chondrocytes in 2D culture and SF-based hydrogels. (n = 3, with three technical replicates)

## Weight and shape retention of gelled biomaterials

To determine the stability of the hydrogels, they were weighed, and their height and diameter were measured over 14 days. The ECM2.5 hydrogel dissolved immediately, when it was put from 4°C to RT, wherefore it was excluded from all analyses. The height of SF2.5, SF5, SF7.5, SF10-ECM7.5 and SF10-ECM10 reduced significantly from day 1 to day 14, while the other samples maintained their height. In general, increasing SF concentrations and ECM concentrations in the SF10-ECM samples led to significantly increased heights (Figure S 5A).

The diameter of all SF and SF10-ECM samples reduced significantly from day 1 to day 14, while the pure ECM samples maintained their diameter. Shrinkage of diameter decreased as a function of increasing SF concentration in pure SF samples for 14 days incubation. Although the mean diameter of SF10 (6.9 cm) was smaller than the ones of the SF10-ECM (7.2 – 8.8 cm) samples on day 1, the difference to the samples with higher ECM concentrations (7.5% and 10% ECM) tended to decrease until day 14 (6.4 cm for SF10 and 6.7 – 7.2 cm for SF10-ECM). The addition of 10% ECM to 10% SF still significantly increased the diameter on all days (Figure S 5B).

The wet weight of the SF and SF10-ECM samples decreased significantly from day 1 to day 14, while the weight of the pure ECM samples increased slightly. The high wet weight losses of the 10% SF samples with increasing ECM concentrations between day 1 and 7 decreased between day 7 and 14. A constant weight loss was observed for the pure SF samples (Figure S 5C). These results indicate that 10% SF resulted in the most stable hydrogels in comparison to lower SF concentrations. Since 7.5% and 10% ECM resulted in a fast reduction of height, diameter and weight, the usage of 2.5% or 5% ECM appeared more suitable.


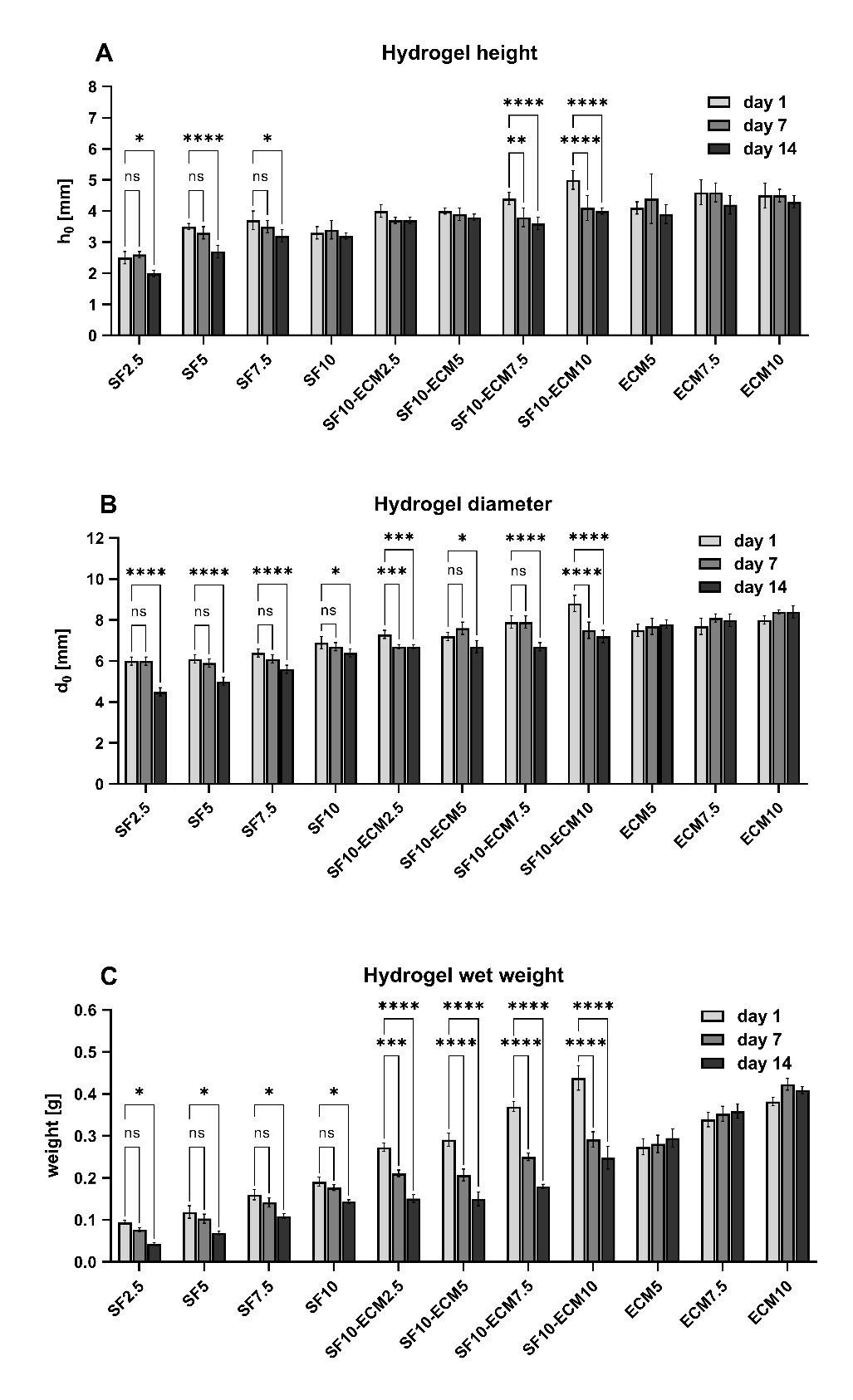


**Figure S 5.** Weight and shape retention of gelled biomaterials for concentration determination on day 1, 7 and 14. The means of three gels as technical replicates are illustrated. A) Hydrogel height. (n = 1) B) Hydrogel diameter. (n = 1) C) Hydrogel wet weight. (n = 1)

<bib id="bib1" type="Periodical"><number>[1]</number>C. Bauer, E. Niculescu-Morzsa, V. Jeyakumar, D. Kern, S. S. Späth, S. Nehrer, *J. Inflamm. (Lond.)* 2016, 13, 31.</bib>

<bib id="bib2" type="Periodical"><number>[2]</number>W. Fu, S. Chen, R. Yang, C. Li, H. Gao, J. Li, X. Zhang, *Elife* 2022, 11, 79585</bib>
